# Comparative genomic analysis of catfish linkage group 8 reveals two homologous chromosomes in zebrafish and other teleosts with extensive inter-chromosomal rearrangements

**DOI:** 10.1186/1471-2164-14-387

**Published:** 2013-06-10

**Authors:** Yu Zhang, Shikai Liu, Jianguo Lu, Yanliang Jiang, Xiaoyu Gao, Parichart Ninwichian, Chao Li, Geoff Waldbieser, Zhanjiang Liu

**Affiliations:** 1 Department of Fisheries and Allied Aquacultures and Program of Cell and Molecular Biosciences, Aquatic Genomics Unit, The Fish Molecular Genetics and Biotechnology Laboratory, Auburn University, Auburn, AL 36849, USA; 2 Catfish Genetics Research Unit, USDA-ARS, 141 Experiment Station Road, Stoneville, Mississippi 38776, USA

**Keywords:** Comparative mapping, Synteny, Genome, Chromosome, Linkage map, Physical map, Catfish, Fish

## Abstract

**Background:**

Comparative genomics is a powerful tool to transfer genomic information from model species to related non-model species. Channel catfish (*Ictalurus punctatus*) is the primary aquaculture species in the United States. Its existing genome resources such as genomic sequences generated from next generation sequencing, BAC end sequences (BES), physical maps, linkage maps, and integrated linkage and physical maps using BES-associated markers provide a platform for comparative genomic analysis between catfish and other model teleost fish species. This study aimed to gain understanding of genome organizations and similarities among catfish and several sequenced teleost genomes using linkage group 8 (LG8) as a pilot study.

**Results:**

With existing genome resources, 287 unique genes were identified in LG8. Comparative genome analysis indicated that most of these 287 genes on catfish LG8 are located on two homologous chromosomes of zebrafish, medaka, stickleback, and three chromosomes of green-spotted pufferfish. Large numbers of conserved syntenies were identified. Detailed analysis of the conserved syntenies in relation to chromosome level similarities revealed extensive inter-chromosomal and intra-chromosomal rearrangements during evolution. Of the 287 genes, 35 genes were found to be duplicated in the catfish genome, with the vast majority of the duplications being interchromosomal.

**Conclusions:**

Comparative genome analysis is a powerful tool even in the absence of a well-assembled whole genome sequence. In spite of sequence stacking due to low resolution of the linkage and physical maps, conserved syntenies can be identified although the exact gene order and orientation are unknown at present. Through chromosome-level comparative analysis, homologous chromosomes among teleosts can be identified. Syntenic analysis should facilitate annotation of the catfish genome, which in turn, should facilitate functional inference of genes based on their orthology.

## Background

Comparative genomics is a powerful tool to transfer genomic information from model species to related non-model species. This approach was first applied to construct a human-chimpanzee comparative genome map using BAC end sequence (BESs) searched against human genome [1]. Subsequently this approach was widely used for comparisons of mammalian genomes such as human-mouse [2], human-cattle [3], human-porcine [4] and human-horse [5] genome comparisons. Recently, comparative genome studies have been conducted in a number of fish species [6-9].

Comparative genomic analyses could bring great benefits to non-model, economically important species. With exception of the recently published cod genome [10], no whole-genome sequence exists for aquaculture fish species. For aquaculture species, comparative genomic analyses not only provide evolutionary perspectives for genome evolution, but also practical applications for the identification of positional candidate genes. It provides a useful tool for genome annotation and functional inference through the analysis of conserved syntenies. This is particularly important because direct testing of functions for a large number of aquaculture species may prove to be difficult to achieve.

Comparative genome analysis requires rich genome resources. With the release of whole genome sequences from five teleost species: zebrafish (*Danio rerio*) (http://www.ensembl.org), fugu (*Fugu rubripes*) [11], green-spotted pufferfish (*Tetraodon nigroviridis*) [12], medaka (*Oryzias latipes*) [13,14] and three-spined stickleback (*Gasterosteus aculeatus*) [15], it is now possible to conduct initial comparative genome analysis for aquaculture species. In recent years, great effort has been devoted for the development of genome resources in aquaculture species. For instance, rich genome resources have been, or are being produced with Atlantic salmon (*Salmo salar*) [16-19], rainbow trout (*Oncorhynchus mykiss*) [20-25], tilapia (*Oreochromis spp*.) [26-31], gilthead sea bream (*Sparus auratus*) [32-34], European sea bass (*Dicentrarchus labrax*) [35-39], and channel catfish (*Ictalurus punctatus*) for reviews, see [40,41]. These genomic resources included expressed sequence tags (ESTs), genetic linkage maps, BAC-based physical maps and radiation hybrid (RH) maps, and draft genome sequences which allow comparative genomic analyses to be conducted. Second, conserved syntenic groups could be established through comparisons of model species with non-model species [42]. The search of conserved syntenies could enhance the identification of gene order, thereby allowing insight into orthologies that may be informative for the analysis of quantitative trait loci (QTL) for commercially important traits [42,43]. In addition, syntenies can provide evolutionary information that support phylogenetic studies for gene and genome annotation [13,42,44].

Channel catfish (*I*. *punctatus*) is the primary aquaculture species in the United States. It is one of the six species included in the U.S. National Animal Genome Project NRSP-8. Major progress has been made in developing genomic resources of catfish. These genomic resources included numerous molecular markers [45-49], genetic linkage maps [50-53], ESTs [54-59], microarray platforms [60-64], transcriptome generated using the next generation sequencing technologies [65-67], BAC libraries [68,69], BAC-based physical maps [70,71], and a partially integrated physical and genetic linkage map [53]. With these genomic resources, comparative genomic analyses were conducted between catfish and model species. Wang *et al*. (2007) utilized 20,366 catfish BESs and identified syntenic regions among the genomes of catfish, zebrafish, and green-spotted pufferfish [69]. In a separate study, Liu *et al*. (2009) compared local conserved syntenies between the catfish and zebrafish genomes using a large number of BAC end sequences [9]. Kucuktas *et al*. (2009) constructed a gene-based catfish linkage map that allowed preliminary comparison of genome similarities among several teleost species [52]. In all these earlier studies, high levels of inter- and intra-chromosomal shuffling were found, suggesting that the generalized linearity relationships may not apply to the organization of the catfish genome when compared to the genomes of other teleosts, as otherwise found between medaka-sea bream, *Tetraodon*-sea bream, stickleback-sea bream, medaka-stickleback, *Tetraodon*-medaka and *Tetraodon*-stickleback genomes [7,42]. However, in these studies, only a small number of gene markers were used that may not allow detection of rearrangement events. Fish-specific genome duplication and accompanying genome rearrangements were reported to lead to teleost species with a higher rate of gene-linkage disruption and lineage divergence than mammals [44,72]. Study on comparison between zebrafish and *Tetraodon* suggested that there were high levels of conserved syntenies between the majority of zebrafish and *Tetraodon* chromosomes, but in the conserved syntenic regions numerous inversions existed involving large regions with altered gene orders and orientations [73]. In this study, we chose catfish linkage group 8 (LG8), which was found to contain microsatellite markers associated with the tolerance to hypoxia (unpublished), as a pilot study to gain greater insight into the similarities and conserved syntenies between the catfish genome and the genomes of several well-characterized fish. Here we report the potential orthologous chromosomes of catfish LG8 in several sequenced fish species, conserved syntenies, annotation of genes on LG8 of the catfish, and identification of a set of duplicated genes.

## Results

### Establishing chromosome-scale scaffolds

In order to conduct comparative genome analysis, the first required step without a whole genome sequence is to establish large scaffolds that can then be compared to chromosomal segments of other species with rich genomic resources. Here, we started with the 106 BAC end sequence-derived microsatellites that were mapped to LG8 [53]. As shown in Table 1, these 106 mapped BAC end sequence-derived microsatellites were from 46 BAC contigs of the physical map [71] that included 1645 BAC end sequences (BESs) [9,48]. Therefore, all these 1645 BESs are on LG8. However, the BESs are short single pass reads and many of them do not contain gene sequences, making their direct comparison with other genomes difficult. Consequently, BLASTN searches using these 1645 BESs against the draft catfish genome sequence contigs (255,858 contigs with N50 of 6027 bp, unpublished data) resulted in 951 significant hits (Table 1).

**Table 1:** **Identification of genes on LG8 using information from BESs**, **the physical map**, **linkage map and draft genome sequences using BLAST analysis**

**Catalog**	**Number**
BAC-associated markers in LG8	106
BAC contigs containing the BAC-associated markers	46
All BAC-end sequences (BES) from mapped BAC contigs	1,645
BESs with significant hits to draft genome	1,510
Draft genome contigs hit by BESs	951
Unique zebrafish genes with genome sequence contig hits	287
Genes with a single genome sequence contig hit	250
Genes with multiple genome sequence contig hits	37

The 951 genome sequence contigs were then used as queries to determine what genes are associated with these genome sequence contigs using BLASTX searches against ENSEMBL zebrafish protein database. The BLASTX searches resulted in 287 unique gene hits. Because the genetic linkage positions of the 1645 BESs are known on LG8, the BLASTX analysis allowed the anchor of the 287 genes on LG8, forming the LG8 scaffold for comparative analysis. Out of the 287 gene hits, 250 genes were hit by a single genome contig while 37 genes were hit by two or more catfish genome contigs (Table 1). The two or more catfish genome sequence contigs that had sequence similarity with a single gene could be from different portion of the same gene (e.g., different exons of the same gene, but yet there are gaps in the draft genome sequence), or from duplicated genes in the catfish genome (see below).

### Identification of homologous chromosomes of catfish LG8

The 287 genes identified on LG8 were used as queries to search the genomes of the four sequenced teleost species, zebrafish, medaka, stickleback, and green-spotted pufferfish. As summarized in Table 2, the largest number of genes had hits on chromosome 7 (148 hits) and chromosome 2 (79 hits) in zebrafish, although significant hits existed for most of the chromosomes, as well as for unassigned scaffolds (Table 2). Similarly, the 287 genes also had largest number of hits on two chromosomes in medaka (chromosome 17 and 18) and stickleback (chromosome 3 and 7), and had largest hits on three chromosomes in green-spotted pufferfish (chromosome 15, 20, and 6). However, green-spotted pufferfish chromosome 1 had 14 gene hits, but there is only one syntenic block involved 2 genes. Therefore green-spotted pufferfish chromosome 1 was not considered as homologous chromosome. These data suggested that the catfish LG8 was homologous to two or three chromosomes in the four sequenced fish genomes (Table 3). As catfish is most closely related to zebrafish phylogenetically, the number of the genes with significant hits was also largest in zebrafish. In green-spotted pufferfish, a large number of these genes have not been assigned to chromosomes, and that is part of the reason that the number of genes with significant hits on the relevant chromosomes was low (Table 2).

**Table 2:** Distributions of LG8 genes on orthologous chromosomes of four model teleost fish species

**Chromosome**	**No**. **of gene hits**			
	**Zebrafish**	**Medaka**	**Stickleback**	**Green**-**spotted pufferfish**
1	6	10	6	14
2	79	1	4	2
3	2	2	53	4
4	3	19	4	4
5	2	6	1	1
6	9	1	2	17
7	148	1	77	5
8	0	4	15	2
9	0	1	12	2
10	0	0	3	10
11	3	1	3	5
12	2	2	2	3
13	0	2	2	2
14	2	5	0	5
15	0	0	12	35
16	1	2	2	0
17	2	51	0	3
18	1	100	5	5
19	2	2	2	1
20	6	30	1	25
21	2	1	29	-
22	1	12	-	-
23	0	2	-	-
24	7	4	-	-
25	2	-	-	-
Unassigned scaffolds	6	13	42	122
Total	287	272	277	267

Chromosomes with a large number of genes of catfish LG8 is underlined.

**Table 3:** Orthologous chromosomes of catfish LG8

**Catfish**	**Zebrafish**	**Medaka**	**Stickleback**	**Green**-**spotted pufferfish**
LG8	Chr 2	Chr 17	Chr 3	Chr 15
	Chr 7	Chr 18	Chr 7	Chr 20 and Chr6

### Annotation of genes on catfish LG8

Annotation in teleost species is often difficult because of the complications caused by gene and genome duplications. Proper annotation of genes from a non-model species requires detailed phylogenetic analysis or analysis of evolutionarily conserved syntenic blocks. Here we have annotated 227 genes on catfish LG8 through comparative analysis of conserved microsyntenies, with 79 genes having significant syntenic conservations on zebrafish chromosome 2 (Additional file 1), and 148 genes having significant syntenic conservations on zebrafish chromosome 7 (Additional file 2 and Additional file 3).

### Conserved syntenic blocks between catfish LG8 and zebrafish

To gain a close insight into the conserved genomic segments, conserved syntenies were examined between the catfish LG8 and zebrafish chromosome 2 and 7. As shown in Additional file 1 and Additional file 2, a total of 37 conserved syntenies were identified. A total of 13 conserved syntenies were identified on chromosome 2 of zebrafish involving 48 genes. These conserved regions span a total of 8.5 million base pairs (Table 4) in the zebrafish genome. Similarly, but to a larger extent, a total of 24 conserved syntenies were identified involving 107 genes on chromosome 7 of zebrafish. These conserved syntenies span a total of 11.2 Mb on zebrafish chromosome 7 (Table 5).

**Table 4:** Summary of conserved syntenic blocks between catfish LG8 and zebrafish chromosome 2

**Syntenic block on zebrafish Chr2**	**Catfish BAC contigs**	**Number of genes**	**Spanning size on zebrafish chr (kb)**
1	Contig2535	2	57
2	Contig0034	2	53
3	Contig 2461	2	42
4	Contig 2732	3	99
5	Contig 1723 (1)	11	1,301
6	Contig 0570	4	2,489
7	Contig 0481	4	381
8	Contig 2727	2	75
9	Contig 0672	4	1,036
10	Contig 1676	5	955
11	Contig 1724	3	1,463
12	Contig 1723 (2)	4	458
13	Contig 1723 (3)	2	55
Total	11	48	8,464

The numbers in parentheses mean the different snyteny within same physical map contig.

**Table 5:** Summary of conserved syntenic blocks between catfish LG8 and zebrafish chromosome 7

**Syntenic block on zebrafish Chr7**	**Catfish physical contigs**	**Number of genes**	**Spanning size on zebrafish chr (kb)**
1	contig1016	3	217
2	contig 0688	8	927
3	contig 2664 (1)	6	483
4	contig 2770 (1)	3	446
5	contig 2664 (2)	11	944
6	contig 1705 (1)	6	456
7	contig 1919 (1)	6	405
8	contig 1705 (2)	4	281
9	contig 1919 (2)	5	614
10	contig 2813	6	313
11	contig 1258	2	185
12	contig 0726	2	146
13	contig 2120	5	400
14	contig 0174	2	373
15	contig 2214	3	189
16	contig 0067 (1)	3	72
17	contig 1919	6	719
18	contig 2770 (2)	7	1,218
19	contig 2665	3	351
20	contig 1918	3	302
21	contig 0067 (2)	4	593
22	contig 1705 (3)	5	550
23	contig 1818	2	244
24	contig 2570	2	753
Total	18	107	11,181

The numbers in parentheses means the different snyteny within same physical contig.

Various lengths of conserved syntenies were identified, ranging from just 40–50 kb to 2.5 Mb (Tables 4 and 5). In some cases, conserved syntenic blocks were extensive involving relatively large number of genes, strongly supporting the syntenic relationships. For instance, catfish contig 1723 was homologous to a genomic segment of 1.3 Mb involving 11 identified genes on zebrafish chromosome 2, and the zebrafish intergenic spaces (without consideration of the gene size) are 350 kb, 41 kb, 73 kb, 199 kb, 15 kb, 66 kb, 65 kb, 215 kb, 98 kb, and 171 kb, indicating linearity relationships of genes and their positions (Additional file 1). In other cases, however, large conserved syntenic blocks were identified involving only a small number of genes, less supportive of linearity relationships. For instance, the largest conserved syntenic block on zebrafish chromosome 2 spans a segment of 2.49 Mb (Table 4), but only four genes are included in the BAC contig 570. The intergenic spaces (without consideration of the gene sizes) were 107 kb, 225 kb, and 2 Mb between them, suggesting a huge deletion within the catfish genome among these genes as compared to the zebrafish genome, or a large number of genes in this region have not been detected in the catfish draft genome sequences.

Conserved syntenic blocks between catfish and medaka, catfish and stickleback and between catfish and green-spotted pufferfish were also conducted (Additional files 3, 4, 5, 6, 7, 8, 9, 10, 11, 12, 13, 14 and 15). The situations are similar to the comparison with the zebrafish genome. Overall, the scale of conserved synteny is largest between catfish LG8 and zebrafish chromosome 7 and chromosome 2, followed by medaka, stickleback, and green-spotted pufferfish (Additional files 3, 4, 5, 6, 7, 8, 9, 10, 11, 12, 13, 14 and 15).

### Chromosome level structural conservations

**Figure 1:**
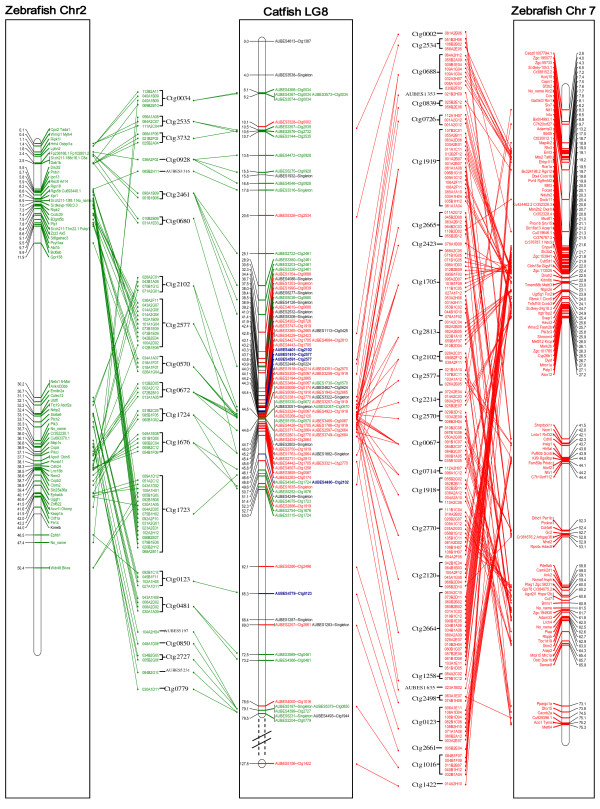
**Comparative map between catfish LG8 and zebrafish chromosome 2 and chromosome 7.** The catfish LG8 is presented in the center panel, and zebrafish chromosome 2 (chr2) and chromosome 7 (chr7) are presented on the left panel and right panel, respectively. For zebrafish chromosomes, gene locations along the chromosome are indicated in Mb on the left (chr2) and right (chr7) of the chromosome(s), while gene names are indicated on the right (chr2) and left (chr7). For catfish LG8, genetic linkage position is indicated in cM on the left, and the gene-associated physical contigs are indicated on the righ. Markers with green labels are associated with genes homologous to zebrafish chr2 and markers with red labels are associated with genes homologous to zebrafish chr7. Markers with blue labels are associated with contigs containing genes homologous to both zebrafish chr2 and chr7.

As described above, BLAST searches indicated that the catfish LG8 is homologous to two chromosomes of zebrafish, medaka, and stickleback, and three chromosomes of green-spotted pufferfish (Table 3). We then focused on the gene position and gene order conservations at the chromosome level. For instance, 148 genes on zebrafish chromosome 7 were determined to be on the catfish LG8. An examination of the chromosome locations of these 148 genes indicated that they were present on zebrafish chromosome 7 at positions from 2.6 Mb to 9.7 Mb, 17 Mb to 27.2 Mb, 41.5 Mb to 44.4 Mb, 52.3 Mb to 53.1 Mb, 58.8 Mb to 65.9 Mb and 73.1 Mb to 75.3 Mb, spanning a physical distance of 30.1 Mb. Without a whole genome assembly in catfish, a complete comparison of gene positions is not yet possible at present because many genes were found to be in each of the physical map contigs, but the resolution of the genetic linkage map that positioned the linked BAC contigs was not high enough to put the catfish genes on a linear order. Therefore, many catfish genes are “stacked”. Nonetheless, we were able to compare the gene positions and order at the chromosome level, ignoring the stacked genes. As shown in Figure 1, homologous genes located on a large segment of zebrafish chromosome 7 of approximately 10.2 Mb (from 17 Mb to 27.2 Mb ) existed on the catfish LG8, spanning a genetic distance of 26 cM. However, this chromosome segment was rearranged in the catfish LG8 in at least 10 major blocks (Figure 1 and Additional file 2). The first block, located on LG8 position 44.5 cM included 6 stacks of genes that are located on zebrafish chromosome 7 at chromosomal location 18.7-19.2 Mb. The second block, located on LG8 position 44.4 cM, included 3 stacks of genes that are located on zebrafish chromosome 7 at location 19.2-19.7 Mb. The third block, located on LG8 position 44.5 cM, included 11 stacks of genes that are located on zebrafish chromosome 7 at location 19.7-20.6 Mb. The fourth block, located on LG8 position 43 cM, included 6 stacks of genes that are located on zebrafish chromosome 7 at location 20.7-21.1 Mb. The fifth block, located on LG8 position 42 cM, included 5 stacks of genes that are located on zebrafish chromosome 7 at location 21.2-21.6 Mb. The sixth block, located on LG8 position 43 cM, included 4 stacks of genes that are located on zebrafish chromosome 7 at location 21.7-22 Mb. The seventh block, located on LG8 position 42 cM, included 5 stacks of genes that are located on zebrafish chromosome 7 at location 22.4-23 Mb. The eighth block, located on LG8 position 43 cM, included 6 stacks of genes that are located on zebrafish chromosome 7 at location 23.8-24 Mb. Another two blocks from 45 cM and 44 cM involved 5 and 3 genes, which spanned 25.9-26.2 Mb and 26.9-27.2 Mb on zebrafish chromosome 7.

Similarly, the 79 zebrafish genes located on two major segments of chromosome 2 spanning a physical distance of 29 Mb on the zebrafish genome, and they were mapped to the catfish LG8 spanning a genetic distance of 15 cM. Very similar to the situation of the comparison between the catfish LG8 with zebrafish chromosome 7, comparison of the catfish LG8 with zebrafish chromosome 2 also revealed extensive chromosome rearrangement in the catfish genome.

Comparative analyses were also conducted between catfish and medaka, catfish and stickleback, catfish and green-spotted pufferfish (Additional files 16, 17 and 18). The situations are highly similar to the comparison with the zebrafish genome. Overall, the organization of the catfish LG8 is most similar to that of zebrafish chromosome 7 and chromosome 2, followed by medaka, stickleback, and green-spotted pufferfish. In addition, comparative map indicated that green-spotted pufferfish chormorsome 15 is homologous to zebrafish 2, but chromosome 20 and 6 are homologous to zebrafish chromosome 7, since the catfish physical contigs with significant gene hits on zebrafish chromosome 7 had significant gene hits on both chromosome 20 and chromosome 6 of green-spotted pufferfish. These findings here are consistent with Woods et al. [72], who reported that *Tetraodon* chromosome 15 is homologous to zebrafish chromosome 2. However, *Tetraodon* chromosome 20 is homologous to zebrafish chromosome 7 and 14, and *Tetraodon* chromosome 6 is homologous to zebrafish chromosome 7, 2, and 24 [72].

### Evolutionary junctions of chromosome rearrangement

Comparisons between syntenic blocks on catfish LG8 and zebrafish chromosome 7 and chromosome 2 (as well as those in medaka, stickleback, and green-spotted pufferfish) indicated extensive chromosomal rearrangements that fused the sequences on two chromosomes together within the catfish genome during evolution. Through sequence analysis, genes involved in the same catfish physical map contig were found to be located on two chromosomes in the zebrafish genome. For instance, 15 genes were identified in the catfish physical map contig 2577 (Additional file 3). Eleven of the 15 genes were found on zebrafish chromosome 2 while four of the 15 genes were found on zebrafish chromosome 7. Similarly, four of the 12 genes in the catfish physical map contig 123 were found on zebrafish chromosome 2 while eight of the 12 genes on zebrafish chromosome 7; four of the 7 genes within the catfish physical map contig 2102 were found on zebrafish chromosome 2 and three genes were on zebrafish chromosome 7 (Additional file 3). Taken together, these findings suggested the presence of fusion junctions in these physical map contigs.

### Duplicated genes on catfish LG8

As discussed above, BLASTX analysis revealed that 37 genes match more than one draft catfish genome sequences (Table 1). These 37 genes are potentially involved in duplicated genes on catfish LG8, though an alternative possibility is that the two or more catfish genome contigs were unassembled contigs in the draft genome assembly. In order to identify the potentially duplicated genes on LG8, all the 287 genes on LG8 were searched against catfish genome sequence contigs. The basic principle is that genes mapped to different genomic locations (e.g., different genome contigs) are potentially duplicated, whereas the careful visual inspection needs to be applied. From BLASTN searches (E-value ≤ 1E-10), a total of 159 genes were hit by multiple genome contigs. Through visual inspections of the homologous regions of these 159 genes, 76 genes match more than one genomic sequence contigs by overlapping regions, suggesting that they may be potentially duplicated genes on LG8 (Table 6). BLASTN searches (cutoff 1E-10) were carried out using the duplication-involved genome contigs to determine if these are truly duplicated genes. A total of 227 genome contigs that potentially represented duplicated genes were used as queries to search against themselves followed by visual inspection of the alignments. A total of 35 genes were identified as duplications on the catfish LG8 (Table 6).

**Table 6:** Summary of duplicated gene identification on LG8

**Catalog**	**Number**
Genes with single gnome contig hit	71
Gene with mutiple genome contig hits, but without overlapping on hitting region	83
Gene with mutiple genome contig and with overlapping on hitting region	76
Potential duplications after analysis using sequence alignments	35

To further determine if these 35 genes were duplicated in the zebrafish genome, web-based BLASTP in ENSEMBL was used to align these 35 genes with zebrafish protein database with genomic locations. A total of 30 (86%) genes out of the 35 genes were determined to be duplicated in the zebrafish genome as well (Table 7).

**Table 7:** A list of duplicated genes on LG8

**Gene ID**	**Gene identity**	**Nature of duplication**	**Duplication in zebrafish**	**Zebrafish chromosome locations**	
ENSDARG00000020857	Coiled-coil domain containing 149b	Inter-chr	+	Chr1: 40,426,579-40,445,530	Chr7: 73,934,667-73,961,321
ENSDARG00000078251	Testis-specific kinase 1	Inter-chr	+	Chr1: 40,601,010-40,619,684	Chr7: 25,695,507-25,738,547
ENSDARG00000011233	Phosphate cytidylyltransferase 1, choline, alpha a	Inter-chr	+	Chr2: 9,544,319-9,572,728	Chr18: 44,426,569-44,436,626
ENSDARG00000011600	Eph receptor a4b	Inter-chr	+	Chr2: 40,081,937-40,266,727	Chr24: 28,418,728-28,585,492
ENSDARG00000014692	Zinc finger protein 622	Inter-chr	-	Chr2: 41,536,204-41,544,585	-
ENSDARG00000014986	Activin a receptor, type i like	Inter-chr	+	Chr2: 41,563,236-41,583,420	Chr23: 28,076,284-28,097,688
ENSDARG00000045137	Histamine receptor h4	Inter-chr	+	Chr2: 1,358,788-1,372,630	Chr22: 1,111,647-1,145,152
ENSDARG00000053293	Fintrim family, member 14	Inter-chr	+	Chr2: 43,232,265-43,236,818	Chr2: 43,804,098-43,806,620
ENSDARG00000054746	Udp-glucose:glycoprotein glucosyltransferase 1	Inter-chr	-	Chr2: 40,583,387-40,642,628	-
ENSDARG00000062991	Abl-interactor 1b	Inter-chr	+	Chr2: 9,651,729-9,704,711	Chr24: 6,018,848-6,096,165
ENSDARG00000067818	Replicase/helicase/endonuclease	Inter-chr	-	Chr3: 44,040,671-44,043,730	-
ENSDARG00000093045	Protein nlrc3-like	Inter-chr	+	Chr4: 49,816,584-49,827,190	Chr1: 37,643,376-37,652,927
ENSDARG00000001241	Poly-u binding splicing factor b	Inter-chr	+	Chr7: 43,876,256-43,887,769	Chr2: 32,371,845-32,389,729
ENSDARG00000032458	Map/microtubule affinity-regulating kinase 2	Inter-chr	+	Chr7: 26,056,874-26,144,251	Chr21: 26,599,159-26,667,051
ENSDARG00000056690	Myotubularin related protein 1a	Inter-chr	+	Chr7: 26,984,248-27,002,780	Chr21: 33,053,927-33,084,023
ENSDARG00000068557	5-hydroxytryptamine (serotonin) receptor 5a like	Inter-chr	+	Chr7: 43,811,481-43,832,125	Chr2: 29,650,026-29,653,812
ENSDARG00000069463	Arachidonate 12-lipoxygenase	Inter-chr	-	Chr7: 27,155,574-27,173,814	-
ENSDARG00000070107	Sine oculis homeobox homolog 7	Inter-chr	+	Chr7: 8,229,740-8,248,372	Chr13: 9,823,203-9,826,579
ENSDARG00000074367	Ubiquitin carboxyl-terminal hydrolase	Inter-chr	+	Chr7: 52,780,107-52,812,002	Chr20: 23,071,313-23,099,240
ENSDARG00000074813	Bloodthirsty-related gene family, member 5	**Intra**-**chr**	+	Chr7: 16,963,727-16,970,800	Chr7: 16,970,942-16,980,218
ENSDARG00000075485	Kinesin light chain 2	Inter-chr	+	Chr7: 7,710,099-7,745,347	Chr22: 41,392,590-41,425,645
ENSDARG00000075647	Grb10 interacting gyf protein 1	Inter-chr	+	Chr7: 22,013,314-22,051,954	Chr7: 22,066,424-22,077,128
ENSDARG00000076302	Deltex homolog 4	Inter-chr	+	Chr7: 19,772,977-19,815,910	Chr1: 41,879,456-41,911,414
ENSDARG00000079906	Calcium channel, voltage-dependent, beta 2a	Inter-chr	+	Chr7: 74,455,736-74,500,031	Chr3: 15,773,660-15,847,471
ENSDARG00000090874	Leukocyte immune-type receptor 3 precursor	Inter-chr	+	Chr7: 7,161,386-7,169,236	Chr7: 6,472,649-6,485,064
ENSDARG00000025789	Chromodomain helicase dna binding protein 4	Inter-chr	+	Chr16: 33,984,640-34,013,093	Chr19: 5,625,090-5,682,362
ENSDARG00000075603	Bloodthirsty-related gene family, member 20	Inter-chr	+	Chr19: 2,844,513-2,855,533	Chr19: 4,855,762-4,867,193
ENSDARG00000016464	Serine/threonine-protein kinase mrck alpha-like	Inter-chr	+	Chr20: 35,110,850-35,230,881	Chr17: 8,164,393-8,331,381
ENSDARG00000017338	Kinase d-interacting substrate of 220b	Inter-chr	+	Chr20: 29,769,812-29,809,552	Chr17: 34,978,387-35,119,673
ENSDARG00000032238	Dynamin 3	Inter-chr	+	Chr20: 14,733,643-14,839,604	Chr5: 66,937,070-67,009,337
ENSDARG00000059933	Phosphatidic acid phosphatase type 2b	Inter-chr	-	Chr20: 8,143,071-8,201,815	-
ENSDARG00000004988	Protein phosphatase 3, catalytic subunit, alpha isozyme	Inter-chr	+	Chr21: 28,352,865-28,470,208	Chr14: 7,770,521-7,857,833
ENSDARG00000078791	General transcription factor ii-i repeat domain-containing protein 2-like	Inter-chr	+	Chr22: 8,348,278-8,349,747	Chr15: 10,383,223-10,384,977
ENSDARG00000052330	Solute carrier family 4, anion exchanger, member 2b	Inter-chr	+	Chr24: 34,531,261-34,600,148	Chr2: 32,109,772-32,175,798
ENSDARG00000071501	Heparan sulfate 6-o-sulfotransferase 1b	Inter-chr	+	Chr24: 28,654,200-28,749,436	Chr9: 30,372,294-30,460,884

## Discussion

In this paper, we present the evidence that the catfish LG8 are homologous to two chromosomes in several sequenced teleost fish species, zebrafish, medaka, and stickleback, and to three chromosomes of green-spotted pufferfish. Such findings were made possible by establishing chromosome level scaffolds using BAC end sequences, the catfish physical map, and the catfish genetic linkage map [9,48,53,71].

Although there are sequence similarities between catfish and zebrafish at various levels, we decided to use only gene sequences for our analysis because gene sequences are more unique and highly conserved in the teleost genomes while sequences from intergenic regions are more divergent, and may involve repeated sequences. Through analysis of 287 genes within the catfish LG8, it is apparent that these genes are located mostly on two or three chromosomes of other teleost species (Table 3). The largest number of genes was found in zebrafish on the two relevant chromosomes with 227 out of 287 genes located on chromosome 2 and chromosome 7, followed by medaka with 151 genes, stickleback with 130 genes, and green-spotted pufferfish with just 77 genes. This is partly due to many of the genes were unassigned to chromosomes with green-spotted pufferfish and Stickleback (Table 2), but is consistent with their phylogenetic relationships with catfish.

Analysis of conserved microsyntenies allowed identification of gene positions and order in different species, thereby establishing potential orthologies. Through analyses of sequence similarity, genome context and neighboring genes, we were able to annotate a relatively large number of genes on catfish LG8. The inferred orthologies are useful for genome annotation in catfish, and perhaps also useful for functional inference. Orthology inference of gene functions will prove to be an effective approach for the vast majority of genes with aquaculture species [74].

The catfish LG8 has a high level of similarity with part of zebrafish chromosome 2 and chromosome 7 (and similarly with two chromosomes in medaka, stickleback and three chromosomes in *Tetraodon*). However, extensive chromosome rearrangement must have occurred. Numerous small syntenic blocks were identified (Additional file 1 and Additional file 2), with some spanning only 40–50 Kb while others spanning well over 2 Mb (Tables 4 and 5). It is apparent that the catfish genome is well conserved at the chromosomal level with those of other teleosts, but extensive local shuffling lead to differences in gene positions and orientations.

Genes included in the same catfish physical map contigs were found on two chromosomes in zebrafish, medaka, stickleback, and green-spotted pufferfish. For instance, genes included in physical map contigs 2577, 123, and 2102 were found to be on chromosome 2 and chromosome 7 in zebrafish. One possibility is that the physical map was wrongly assembled due to duplicated genomic segments. However, this possibility did not hold because genetic linkage mapping of the BAC end-associated microsatellites within these contigs placed the BAC clones on the same linkage group, LG8. In addition, we have examined the physical map assembly with extremely high stringencies at p = 10^-40^, the associated genes from the same BAC contigs still had hits to genes on both chromosome 2 and chromosome 7 in zebrafish. Furthermore, in some cases two genes on the same catfish BAC clone are homologous to genes on two different zebrafish chromosomes. For instance, the two genes from mate paired reads of BAC end sequences residing within ctg2102 were homologous to “cadherin 24, type 2” located on zebrafish chromosome 2, and to “mannose receptor, C type 1a” located on zebrafish chromosome 7 (Additional file 3). Taken together, these physical contigs should harbor the fusion junctions of the sequences from the two chromosomes during evolution. Analysis of such junctions is not possible at present because the sequences are not yet available, but it should be interesting to look into these junctions to reveal evolutionary events in forming the chromosome represented by LG8.

It is interesting to observe a higher level of genome scale structural conservation between catfish and zebrafish than between catfish and the other three fish species. However, it is also intriguing that catfish has 29 chromosomes whereas zebrafish has 25, medaka has 24, stickleback has 21, and green-spotted pufferfish has 21 chromosomes, but yet the homologous chromosome segments of LG8 of catfish are distributed on two or three chromosomes in these fish, suggesting that some catfish chromosomes may have to be large to contain genes from several chromosome equivalents of the model fish species, or that significant chromosomal rearrangements have occurred during evolution, in contrast to the generalized linearity relationships among medaka, stickleback, green-spotted pufferfish, and sea bream as previously reported [42]. To the minimum, it is expected that in some cases, one chromosome of zebrafish (and more so with medaka, stickleback and *Tetraodon* because they have even fewer chromosomes) should be equivalent to more than one catfish chromosomes. Whole genome comparative mapping is warranted to address such issues.

After two rounds of whole genome duplication in vertebrates, ray-finned fishes (actinopterygian) had a third round, fish-specific genome duplication ~350 million years ago (FSGD or 3R) [12,72,75,76]. Studies on Hox gene clusters from a spectrum of vertebrate species provided critical evidence in support of this hypothesis [77,78]. In addition, several studies have suggested increased rate of inter-chromosomal rearrangements following the whole-genome duplication (WGD) [13,44,79]. Further studies suggested eight major interchromosomal rearrangements in the 24 ancestor chromosomes in teleosts [13]. Subsequently, the medaka lineage preserved its ancestral genomic structure and green-spotted pufferfish lineage underwent three major rearrangements, while the zebrafish lineage has experienced many interchromosomal rearrangements [13]. From the comparison of chromosome blocks among the five teleost species under study, it is apparent that many inter- as well as intra-chromosomal rearrangements may have occurred.

However, the conserved syntenies we identified between catfish LG8 and zebrafish chromosomes 2 and 7, and medaka chromosomes 17 and 18 are consistent with the ancestral vertebrate linkage groups model presented by Nakatani et al. [80] and Danzmann et al. [81]. According to that model, there is strong affinity between the ancestral chromosome M and zebrafish chromosome 2 and medaka chromosome 17. Similarly, there is partial affinity between the ancestral chromosome F and zebrafish chromosome 7 and medaka chromosome 18. Our results here provide additional support to the ancestral chromosome model, and hold promise for whole-genome comparative genome analysis.

A set of potentially duplicated genes were identified by sequence alignment analysis. Although the final status of the nature of duplication requires additional work, particularly the sequence assembly of the whole genome sequence, it is apparent that 35 out of 287 (12.2%) genes on catfish LG8 were duplicated. This rate of gene duplication is similar to that found in zebrafish genome (14.9%) [82]. In addition Bloodthirsty-related gene family member 5 and its duplicated copy are located on the same scaffold in catfish, suggesting that they are intra-chromosomal duplication in the catfish genome. Interestingly, this duplication pair is also located on the same chromosome in zebrafish (Table 7). Other 34 putative duplicated genes are potentially inter-chromosomal duplication because they are located on different scaffolds that have been mapped to different linkage groups [53]. Therefore, all but one of the 35 duplicated genes are inter-chromosomal, consistent with the situations in related teleost species [82].

## Conclusion

In this study, integrated genome resources with BAC end sequences, physical map, linkage map and the draft genome sequences were used to conduct comparative genome analysis of the catfish LG8. The catfish LG8 was found to be homologous to two chromosomes in zebrafish, medaka, stickleback and three chromosomes in green-spotted pufferfish. Through syntenic analysis, a large number of genes were annotated on LG8. Detailed analysis of syntenic blocks suggested extensive inter- and intra-chromosomal rearrangements in the catfish genome, with certain BAC contigs identified to contain evolutionary fusion junctions. A set of potentially duplicated genes was identified. As a pilot project, this work provided the proofs of the principle for whole genome comparative mapping, and for whole genome sequence assembly and annotation.

## Methods

### Establishing chromosome-scale scaffolds

**Figure 2:**
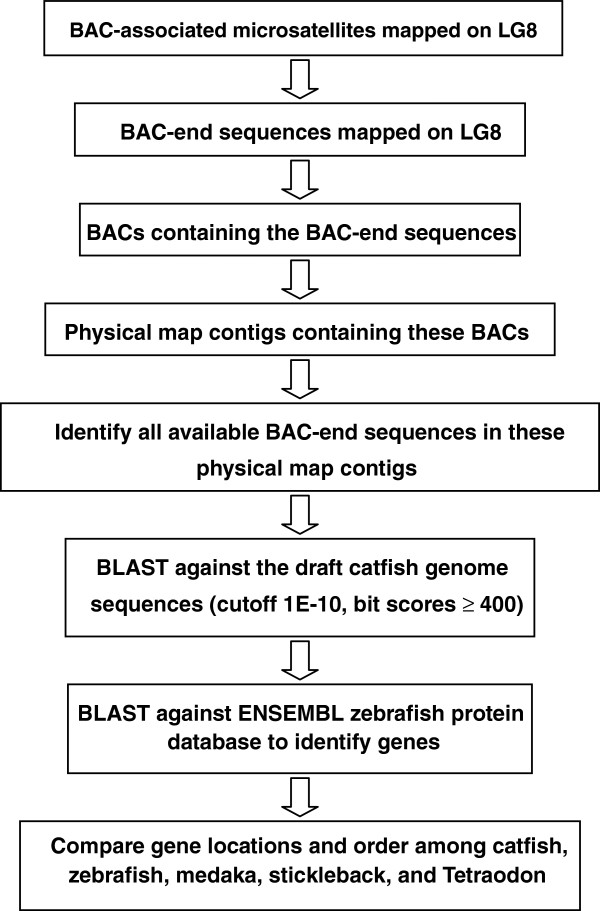
**Flowchart of establishing chromosome**-**scale scaffolds.**

The flow chart of this work is illustrated in Figure 2. This work started with genetically mapped BAC end sequences using microsatellite markers [53], the catfish physical map [71], the BAC end sequences, and the draft catfish genome sequence contigs (unpublished data). The BAC end sequences were previously described and they were deposited to GenBank [9,48]. The basic concept is that when one BAC end sequence is mapped to LG8, the entire BAC contig is mapped to LG8. BAC clones within the same BAC contigs as the mapped BAC clones were identified through the examination of the catfish physical map [71]. All available BAC end sequences within the BAC contigs were then collected from the NCBI database. A total of 1,645 BAC end sequences were obtained and used to conduct BLAST searches against the draft catfish genome sequence database with E-value ≤1E-10. The genome sequence contigs that were “mapped” to LG8 were filtered with high stringent bit scores ≥ 400 to ensure the identification of true homologous sequences.

### Gene identification on LG8

The mapped genome sequence contigs were repeat-masked using RepeatMasker (version 3.2.7, http://www.repeatmasker.org/) to mask repetitive sequences before the BLASTX search for gene identification. The repeat-masked genome sequence contigs were used as queries for BLASTX search against the ENSEMBL zebrafish protein database (Danio rerio Zv9.67) with an E-value cutoff of 1E-10. Gene annotation information was retrieved by BioMart (http://www.biomart.org) with ENSEMBL gene IDs. For uncharacterized genes in ENSEMBL, BLAST search was conducted against NCBI nr database to obtain the gene annotation information.

### Identification of homologous chromosomes

The homologous chromosomes and gene locations in zebrafish were obtained using BioMart with the unique ENSEMBL gene IDs. For medaka, stickleback and green-spotted pufferfish, similarly, BLASTX searches were conducted using gene-coding sequences. The coding sequences as query were searched against protein databases: medaka (MEDAKA1.68), stickleback (BROADS1.68) and green-spotted pufferfish (TETRAODON8.68) with the E-value cutoff of 1E-10, respectively. The homologous chromosomes and gene locations were then identified by BioMart. Homologous chromosomes were identified as the chromosomes with high number of gene hits.

### Identification of conserved syntenies

Conserved syntenies were identified based on genetic positions of BAC end associated microsatellite markers, the associated genes on the linkage map and model fish chromosomal locations. Putative conserved syntenies were established when the genes were located in the same chromosome and the same linkage group. Microsyntenic blocks were identified based on genes included within BAC contigs of the catfish physical map and their locations on one chromosome of model fish. The putative conserved microsyntenies were identified as segments of model fish chromosomes with a set of adjacent genes that are homologous to a set of adjacent genes in catfish that are reflected by their colocation within a single BAC contig. For the BAC contigs with significant hits on more than one model fish chromosomes, e.g., ctg0123, ctg2577 and ctg2102 were mapped on both zebrafish chromosome 7 and chromosome 2, the physical maps with high stringent cutoff value: 10^-40^, 10^-30^ and 10^-25^ were checked to determine if these BAC contigs were incorrectly assembled in the physical map which was constructed using a cutoff value of 10^-20^[71].

Comparative maps were constructed by using MapChart [83]. The BAC contigs were anchored to the linkage group based on the BES-associated microsatellite markers. The comparative maps were then drawn based on the positions of BAC contigs on catfish LG8 and the gene locations on model fish chromosomes.

### Analysis of gene duplication on LG8

All the 287 genes on LG8 were used as queries to search against catfish whole genome sequence assembly (unpublished data) to identify potential duplicated genes. Theoretically, the genes with significant hits to different genomic regions (e.g., different genome contigs) should represent duplicated genes. However, the current catfish genome assembly is still incomplete. Therefore, the genes with hits of multiple genomic contigs were used as a starting point for further analysis and visual inspections. All the catfish genome contigs involved in potential duplications were retrieved and visually checked by sequence alignments using BLASTN at a cutoff value of 1E-10 and minimum alignment length of 100 bp. The nature of duplicated genes were determined by examination of their genomic locations, with the understanding that if they are located in the same contig or scaffold, then the duplicated genes are tandem or intra-chromosome, but not inter-chromosome. In contrast, if they are located in different scaffolds, they are candidates for inter-chromosomal duplications, pending mapping of the two scaffolds to different chromosomes.

To determine if the duplicated genes in the catfish genome are also duplicated in the zebrafish genome, duplicated genes in catfish were used as queries to search against the ENSEMBL zebrafish protein database using the Web based ENSEMBL BLAST (cutoff of 1E-10) to determine the genomic locations and coordinates of these genes. The hits with high stringencies (alignment score ≥ 1000) were considered as duplications.

## Competing interests

The authors declare that they have no competing interests.

## Authors' contributions

YZ conducted the major part of the research including data analysis and manuscript preparation. SL provided assistance for data analysis and manuscript preparation. JL, YJ, XG, PN and CL provided help with data analysis. GW involved in the generation of catfish genome resources. ZL supervised the entire study and provided assistance for data analysis and manuscript preparation. All authors read and approved the final manuscript.

## Supplementary Material

Additional file 1 **Annotation of catfish genes mapped in LG8 with significant hits to zebrafish chromosome 2.** Microsyntenies are indicated by the same colored rows. Click here for file

Additional file 2 **Annotation of catfish genes mapped in LG8 with significant hits to zebrafish chromosome 7.** Microsyntenies are indicated by the same colored rows. Click here for file

Additional file 3 Annotation of catfish genes mapped in one physical contig in LG8 with significant hits to both zebrafish chromosome 7 and chromosome 2. Click here for file

Additional file 4 **Catfish genes mapped in LG8 with significant hits to Medaka chromosome 17.** Microsyntenies are indicated by the same colored rows. Click here for file

Additional file 5 **Catfish genes mapped in LG8 with significant hits to medaka chromosome 18.** Microsyntenies are indicated by the same colored rows. Click here for file

Additional file 6 **Summary of conserved syntenic blocks between catfish LG8 and medaka chromosome 17.** The number in parentheses mean the different snyteny within same physical contig. Click here for file

Additional file 7 **Summary of conserved syntenic blocks between catfish LG8 and medaka chromosome 18.** The number in parentheses mean the different snyteny within same physical contig. Click here for file

Additional file 8 **Catfish genes mapped in LG8 with significant hits to stickleback chromosome 3.** Microsyntenies are indicated by the same colored rows. Click here for file

Additional file 9 **Catfish genes mapped in LG8 with significant hits to stickleback chromosome 7.** Microsyntenies are indicated by the same colored rows. Click here for file

Additional file 10 **Summary of conserved syntenic blocks between catfish LG8 and stickleback chromosome 3.** The number in parentheses mean the different snyteny within same physical contig. Click here for file

Additional file 11 **Summary of conserved syntenic blocks between catfish LG8 and stickleback chromosome 7.** The number in parentheses mean the different snyteny within same physical contig. Click here for file

Additional file 12 **Catfish genes mapped in LG8 with significant hits to green-spotted pufferfish chromosome 15.** Microsyntenies are indicated by the same colored rows. Click here for file

Additional file 13 **Catfish genes mapped in LG8 with significant hits to green-spotted pufferfish chromosome 20 and chromosome 6.** Microsyntenies are indicated by the same colored rows. Click here for file

Additional file 14 **Summary of conserved syntenic blocks between catfish LG8 and green-spotted pufferfish chromosome 15.** The number in parentheses mean the different snyteny within same physical contig. Click here for file

Additional file 15 **Summary of conserved syntenic blocks between catfish LG8 and green-spotted pufferfish chromosome 20 and chromosome 6.** The number in parentheses mean the different snyteny within same physical contig. Click here for file

Additional file 16 Comparative map between catfish LG8 and medaka chromosome 17 and chromosome 18. Click here for file

Additional file 17 Comparative map between catfish LG8 and stickleback chromosome 3 and chromosome 7. Click here for file

Additional file 18 Comparative map between catfish LG8 and green-spotted pufferfish chromosome 15, chromosome 20 and chromosome 6. Click here for file
