# Supplementary material for: Comparative genomic analysis of catfish linkage group 8 reveals two homologous chromosomes in zebrafish and other teleosts with extensive inter-chromosomal rearrangements
Source: BMC Genomics. 2013 Jun 10;14:387. doi: 10.1186/1471-2164-14-387 (PMC3691659; doi:10.1186/1471-2164-14-387)
Supplement: Additional file 1 — Annotation of catfish genes mapped in LG8 with significant hits to zebrafish chromosome 2. Microsyntenies are indicated by the same colored rows. [file 1471-2164-14-387-S1.docx]

### S Table 1 - Annotation of catfish genes mapped in LG8 with significant hits to zebrafish chromosome 2. Microsyntenies deteccted are indicated by the same colored rows.

| **BAC contig ID** | **Gene ID** | **Gene Start** | **Description** |
| --- | --- | --- | --- |
| Contig2535 | ENSDARG00000063518 | 93,928 | Carboxypeptidase N, polypeptide 2 |
| Contig2535 | ENSDARG00000088857 | 115,939 | Transcriptional adaptor 1 |
| Contig0034 | ENSDARG00000005218 | 394,830 | Werner helicase interacting protein 1 |
| Contig0034 | ENSDARG00000091260 | 427,011 | Myosin light chain kinase family, member 4 |
| Contig0928 | ENSDARG00000006677 | 1,088,190 | Receptor (TNFRSF)-interacting serine-threonine kinase 1, like |
| Contig2461 | ENSDARG00000045137 | 1,358,788 | Histamine receptor H4 |
| Contig2461 | ENSDARG00000053746 | 1,386,360 | Oxysterol binding protein-like 1A |
| Contig0680 | ENSDARG00000087965 | 1,626,935 | Latrophilin 2 |
| Contig0034 | ENSDARG00000076426 | 2,392,229 | TPA: endonuclease-reverse transcriptase |
| Contig0680 | ENSDARG00000087364 | 2,428,046 | Transposase |
| Contig2732 | ENSDARG00000077288 | 2,490,000 | Si:ch211-188c16.1 |
| Contig2732 | ENSDARG00000039516 | 2,507,005 | Complement component 8, alpha polypeptide |
| Contig2732 | ENSDARG00000003290 | 2,547,314 | Disabled homolog 1b |
| Singleton | ENSDARG00000033259 | 5,486,084 | DIS3 mitotic control homolog-like 2 |
| Contig1723 | ENSDARG00000036106 | 6,240,288 | Regulator of G-protein signaling 18 |
| Contig1723 | ENSDARG00000017860 | 6,590,676 | Regulator of G-protein signaling 5b |
| Contig1723 | ENSDARG00000058701 | 6,631,445 | Carboxyl-terminal PDZ ligand of neuronal nitric oxide |
| Contig1723 | ENSDARG00000029671 | 6,704,193 | Xenotropic and polytropic retrovirus receptor 1 |
| Contig1723 | ENSDARG00000093342 | 6,903,525 | Si:ch211-13f8.1 |
| Contig1723 | ENSDARG00000078299 | 6,918,043 | Procollagen galactosyltransferase 2 |
| Contig1723 | ENSDARG00000093159 | 6,984,391 | Si:dkeyp-106c3.3 |
| Contig1723 | ENSDARG00000095239 | 7,049,396 | Si:dkeyp-106c3.1 |
| Contig1723 | ENSDARG00000033713 | 7,264,822 | Receptor-interacting serine-threonine kinase 2 |
| Contig1723 | ENSDARG00000016485 | 7,362,320 | Coiled-coil domain containing 39 |
| Contig1723 | ENSDARG00000004396 | 7,533,735 | UDP-glcnac:betagal beta-1,3-N-acetylglucosaminyltransferase 5b |
| Contig0570 | ENSDARG00000011233 | 9,544,319 | Phosphate cytidylyltransferase 1, choline, alpha a |
| Contig0570 | ENSDARG00000062991 | 9,651,729 | Abl-interactor 1b |
| Contig0570 | ENSDARG00000069295 | 9,876,775 | B-cell CLL/lymphoma 6a, genome duplicate b |
| Contig0570 | ENSDARG00000079665 | 11,899,842 | G protein-coupled receptor 158 |
| Contig0481 | ENSDARG00000038780 | 32,075,481 | Upstream binding transcription factor, like |
| Contig0481 | ENSDARG00000074435 | 32,193,669 | Tetratricopeptide repeat domain 19 |
| Contig0481 | ENSDARG00000038785 | 32,223,429 | ATP-binding cassette, sub-family F (GCN20), member 2a |
| Contig0481 | ENSDARG00000032951 | 32,393,877 | Nuclear receptor binding protein 2 |
| Contig0779 | ENSDARG00000018534 | 33,064,484 | Solute carrier family 6, member 9 |
| Singleton | ENSDARG00000055026 | 33,685,224 | Patched 2 |
| Contig0850 | ENSDARG00000038754 | 35,324,581 | Polo-like kinase 3 |
| Singleton | ENSDARG00000068288 | 35,714,224 | Laminin subunit gamma-2 |
| Contig2727 | ENSDARG00000076803 | 36,045,319 | Reverse transcriptase-like protein |
| Contig2727 | ENSDARG00000086959 | 36,120,139 | T-cell receptor alpha variable region |
| Contig0672 | ENSDARG00000060326 | 36,641,517 | Microtubule-associated protein 1S |
| Contig0672 | ENSDARG00000004173 | 36,897,687 | Coatomer protein complex, subunit alpha |
| Contig0672 | ENSDARG00000021225 | 37,086,384 | Protein kinase C, iota |
| Contig0672 | ENSDARG00000007425 | 37,671,471 | Apolipoprotein L, 1 |
| Contig1676 | ENSDARG00000086990 | 38,508,419 | Leucine rich repeat containing 16B |
| Contig1676 | ENSDARG00000074064 | 38,629,171 | RAS (RAD and GEM)-like GTP binding 2 |
| Contig1676 | ENSDARG00000038743 | 38,701,553 | Coatomer protein complex, subunit beta 2 |
| Contig1676 | ENSDARG00000060637 | 38,812,535 | Calsyntenin 2 |
| Contig1676 | ENSDARG00000038731 | 39,435,480 | Solute carrier family 25, member 36a |
| Contig1724 | ENSDARG00000011600 | 40,081,937 | Eph receptor A4b |
| Contig1724 | ENSDARG00000054746 | 40,583,387 | UDP-glucose:glycoprotein glucosyltransferase 1 |
| Contig1724 | ENSDARG00000014692 | 41,536,204 | Zinc finger protein 622 |
| Contig1723 | ENSDARG00000014986 | 41,563,896 | Activin A receptor, type I like |
| Contig1723 | ENSDARG00000040306 | 41,589,199 | Otolith matrix protein |
| Contig1723 | ENSDARG00000016132 | 41,936,143 | Kelch-like ECH-associated protein 1a |
| Contig1723 | ENSDARG00000024365 | 41,977,713 | Cytokine receptor-like factor 1a |
| Contig2535 | ENSDARG00000053293 | 43,232,265 | Fintrim family, member 14 |
| Contig2664 | ENSDARG00000056998 | 44,023,127 | Kin of IRRE like b |
| Contig0034 | ENSDARG00000090419 | 47,440,933 | Cell surface flocculin |
| Contig1723 | ENSDARG00000038543 | 50,376,130 | WD repeat domain 48 |
| Contig1723 | ENSDARG00000059857 | 50,414,868 | Biliverdin reductase A |
