# Supplementary material for: Comparative genomic analysis of catfish linkage group 8 reveals two homologous chromosomes in zebrafish and other teleosts with extensive inter-chromosomal rearrangements
Source: BMC Genomics. 2013 Jun 10;14:387. doi: 10.1186/1471-2164-14-387 (PMC3691659; doi:10.1186/1471-2164-14-387)
Supplement: Additional file 2 — Annotation of catfish genes mapped in LG8 with significant hits to zebrafish chromosome 7. Microsyntenies are indicated by the same colored rows. [file 1471-2164-14-387-S2.docx]

### S Table 2. Annotation of catfish genes mapped in LG8 with significant hits to zebrafish chromosome 7. Microsyntenies deteccted are indicated by the same colored rows.

| **BAC contig ID** | **Gene ID** | **Gene Start** | **Description** |
| --- | --- | --- | --- |
| Contig2214 | ENSDARG00000088655 | 2,637,136 | YadA domain-containing protein |
| Contig2732 | ENSDARG00000070852 | 3,975,903 | zgc:195077 |
| Contig1016 | ENSDARG00000094374 | 6,472,649 | Si:dkey-10h3.1 |
| Contig2534 | ENSDARG00000090874 | 7,161,386 | Leukocyte immune-type receptor TS32.15 L1.1a |
| Contig1016 | ENSDARG00000090815 | 7,388,888 | Potassium inwardly-rectifying channel, subfamily J, member 10 |
| Contig1016 | ENSDARG00000045199 | 7,489,102 | Calpain 1, (mu/I) large subunit |
| Contig1016 | ENSDARG00000018049 | 7,561,474 | Splicing factor 3b, subunit 2, 145kda |
| Contig0688 | ENSDARG00000090684 | 7,672,451 | Reverse transcriptase |
| Contig0688 | ENSDARG00000075485 | 7,710,099 | Kinesin light chain 2 |
| Contig0688 | ENSDARG00000092003 | 7,809,067 | Copper chaperone for superoxide dismutase |
| Contig0688 | ENSDARG00000073728 | 8,066,739 | Galactose-3-O-sulfotransferase 3 |
| Contig0688 | ENSDARG00000070100 | 8,140,745 | Ras and Rab interactor 1 |
| Contig0688 | ENSDARG00000070107 | 8,229,740 | Sine oculis homeobox homolog 7 |
| Contig0688 | ENSDARG00000070116 | 8,323,082 | Nitrilase 1 |
| Contig0688 | ENSDARG00000077639 | 8,542,822 | Inturned planar cell polarity effector homolog |
| Contig1016 | ENSDARG00000070065 | 8,847,563 | Leukocyte immune-type receptor |
| Contig0839 | ENSDARG00000076239 | 9,701,566 | Chromosome 20 open reading frame 27 |
| Contig2665 | ENSDARG00000027903 | 13,209,460 | ADAMTS-like 3 |
| Contig2534 | ENSDARG00000074813 | 16,963,727 | Bloodthirsty-related gene family, member 5 |
| Contig2664 | ENSDARG00000088036 | 18,732,725 | Uncharacterized protein |
| Contig2664 | ENSDARG00000088706 | 18,899,304 | Mitogen-activated protein kinase kinase kinase kinase 2 |
| Contig2664 | ENSDARG00000058028 | 18,963,210 | Reticulon 3 |
| Contig2664 | ENSDARG00000058038 | 19,063,331 | Echinoderm microtubule associated protein like 3 |
| Contig2664 | ENSDARG00000013031 | 19,154,527 | Metastasis associated 1 family, member 2 |
| Contig2664 | ENSDARG00000079684 | 19,200,553 | TAF6-like RNA polymerase II, p300/CBP-associated factor (PCAF)-associated factor |
| Contig2770 | ENSDARG00000057353 | 19,239,543 | EH domain binding protein 1-like 1 |
| Contig2770 | ENSDARG00000077909 | 19,450,362 | RCE1 homolog, prenyl protein peptidase a |
| Contig2770 | ENSDARG00000086318 | 19,676,949 | Zinc finger protein 16-like |
| Contig2664 | ENSDARG00000009610 | 19,689,263 | Regulator of G-protein signaling 12 |
| Contig2664 | ENSDARG00000076302 | 19,772,977 | Deltex homolog 4 |
| Contig2664 | ENSDARG00000008660 | 19,832,691 | Coronin, actin binding protein, 1B |
| Contig2664 | ENSDARG00000077419 | 19,871,792 | Poliovirus receptor-related 4 |
| Contig2664 | ENSDARG00000036678 | 19,919,819 | RNA (guanine-9-) methyltransferase domain containing 2 |
| Contig2664 | ENSDARG00000006754 | 19,994,550 | Myeloid/lymphoid or mixed lineage-leukemia translocation to 3 homolog |
| Contig2664 | ENSDARG00000022020 | 20,098,272 | Focadhesin |
| Contig2664 | ENSDARG00000007526 | 20,254,861 | NADH dehydrogenase (ubiquinone) Fe-S protein 2 |
| Contig2664 | ENSDARG00000062485 | 20,393,171 | Dedicator of cytokinesis 11 |
| Contig2664 | ENSDARG00000036575 | 20,558,899 | Probable imidazolonepropionase |
| Contig2664 | ENSDARG00000088656 | 20,588,427 | Uncharacterized protein |
| Contig1705 | ENSDARG00000079463 | 20,667,640 | Mannosidase, alpha, class 2B, member 2 |
| Contig1705 | ENSDARG00000069313 | 20,746,204 | Oxidase (cytochrome c) assembly 1-like |
| Contig1705 | ENSDARG00000089432 | 20,772,655 | Probable E3 ubiquitin-protein ligase RNF144A-A |
| Contig1705 | ENSDARG00000069326 | 21,008,521 | MUS81 endonuclease homolog |
| Contig1705 | ENSDARG00000088810 | 21,088,169 | Prospero-related homeobox gene 1b |
| Contig1705 | ENSDARG00000020397 | 21,112,106 | Sorting nexin 15 |
| Contig1918 | ENSDARG00000055342 | 21,199,624 | Solute carrier family 16, member 13 |
| Contig1919 | ENSDARG00000056346 | 21,216,449 | Arfgap with coiled-coil, ankyrin repeat and PH domains 1 |
| Contig1919 | ENSDARG00000036414 | 21,305,107 | Insulin receptor substrate 1-B-like |
| Contig1919 | ENSDARG00000077414 | 21,492,964 | Putative G-protein-coupled receptor |
| Contig1919 | ENSDARG00000036420 | 21,551,024 | Uncharacterized protein |
| Contig1919 | ENSDARG00000036422 | 21,590,796 | Netrin 3 |
| Contig1919 | ENSDARG00000055797 | 21,617,267 | Canopy4 |
| Contig1705 | ENSDARG00000055791 | 21,693,465 | Solute carrier family 3 |
| Contig1705 | ENSDARG00000069376 | 21,814,538 | Vomeronasal type-2 receptor 1-like |
| Contig1705 | ENSDARG00000056007 | 21,931,439 | Cytochrome b5 domain containing 1 |
| Contig1705 | ENSDARG00000036463 | 21,968,690 | Claudin 15a |
| Contig2423 | ENSDARG00000075647 | 22,013,314 | GRB10 interacting GYF protein 1 |
| Contig1919 | ENSDARG00000036445 | 22,441,345 | Serpin H1-like |
| Contig1919 | ENSDARG00000087352 | 22,495,105 | Dynein, axonemal, heavy chain 2 |
| Contig1919 | ENSDARG00000088087 | 22,933,203 | Lysine (K)-specific demethylase 6B |
| Contig1919 | ENSDARG00000069388 | 22,955,984 | Transmembrane protein 88 b |
| Contig1919 | ENSDARG00000012827 | 23,045,025 | Methyltransferase like 3 |
| Contig1422 | ENSDARG00000077329 | 23,113,239 | Neuroligin 2a |
| Contig2813 | ENSDARG00000032862 | 23,859,052 | UDP glucuronosyltransferase 5 family, polypeptide G1 |
| Contig2813 | ENSDARG00000016260 | 23,885,650 | Fragile X mental retardation, autosomal homolog 2 |
| Contig2813 | ENSDARG00000036386 | 23,968,556 | RNA binding motif protein 4.1 |
| Contig2813 | ENSDARG00000022466 | 24,022,206 | Chloride channel 5 |
| Contig2813 | ENSDARG00000004196 | 24,081,951 | Tumor necrosis factor (ligand) superfamily, member 10 like |
| Contig2813 | ENSDARG00000034855 | 24,149,053 | Cyclin B3 |
| Contig1258 | ENSDARG00000036560 | 24,728,126 | Si:dkey-24g18.3 |
| Contig1258 | ENSDARG00000030176 | 24,894,860 | Integrin beta 1 binding protein (melusin) 2 |
| Contig0726 | ENSDARG00000013526 | 25,419,150 | Sushi, von Willebrand factor type A, EGF and pentraxin domain containing 1 |
| Contig0726 | ENSDARG00000061385 | 25,558,030 | HAUS augmin-like complex, subunit 3 |
| Contig0839 | ENSDARG00000061411 | 25,661,328 | Wolf-Hirschhorn syndrome candidate 2 |
| Singleton | ENSDARG00000054786 | 25,675,214 | Fatty acid amide hydrolase 2b |
| Contig0726 | ENSDARG00000054829 | 25,760,357 | Phosphoinositide-3-kinase, class 3 |
| Contig2120 | ENSDARG00000079900 | 25,896,649 | Shroom family member 4 |
| Contig2120 | ENSDARG00000054856 | 25,995,961 | Methyltransferase like 12 |
| Contig2120 | ENSDARG00000040224 | 26,048,763 | Kelch repeat-containing protein |
| Contig2120 | ENSDARG00000032458 | 26,056,874 | MAP/microtubule affinity-regulating kinase 2 |
| Contig2120 | ENSDARG00000054934 | 26,293,720 | Glyoxal reductase |
| Contig0174 | ENSDARG00000077121 | 26,490,839 | Cytochrome P450, family 26, subfamily b, polypeptide 1 |
| Contig0174 | ENSDARG00000007370 | 26,796,246 | Dysferlin, limb girdle muscular dystrophy 2B |
| Contig2214 | ENSDARG00000056690 | 26,984,248 | Myotubularin related protein 1a |
| Contig2214 | ENSDARG00000079313 | 27,132,788 | Proline, glutamate and leucine rich protein 1 |
| Contig2214 | ENSDARG00000069463 | 27,155,574 | Arachidonate 12-lipoxygenase |
| Contig0002 | ENSDARG00000059871 | 41,451,484 | Sharpin and rbck1 related |
| Contig2534 | ENSDARG00000060027 | 42,037,543 | Nucleolar protein with MIF4G domain 1 |
| Contig2661 | ENSDARG00000037478 | 42,291,449 | Cadherin 8, type 2 |
| Singleton | ENSDARG00000068557 | 43,811,481 | 5-hydroxytryptamine (serotonin) receptor 5A like |
| Contig2498 | ENSDARG00000090618 | 44,364,516 | Chromosome 10 open reading frame 112 |
| Contig0067 | ENSDARG00000086452 | 52,257,081 | Death domain containing 1 |
| Contig0067 | ENSDARG00000012499 | 52,277,645 | Period homolog 1b |
| Contig0067 | ENSDARG00000052057 | 52,304,510 | Procollagen C-endopeptidase enhancer |
| Contig1919 | ENSDARG00000052061 | 52,375,619 | Collagen, type IV, alpha 6 |
| Contig1919 | ENSDARG00000018329 | 52,724,991 | Guanylyl cyclase 2 |
| Contig1919 | ENSDARG00000074367 | 52,780,107 | Ubiquitin carboxyl-terminal hydrolase |
| Contig1919 | ENSDARG00000059672 | 52,820,279 | Rho gtpase activating protein 36 |
| Contig1919 | ENSDARG00000089066 | 52,927,886 | NHS-like 2 |
| Contig1919 | ENSDARG00000014690 | 53,088,995 | Ribosomal protein S4, X-linked |
| Contig2120 | ENSDARG00000003021 | 53,111,289 | Histone deacetylase 8 |
| Contig2770 | ENSDARG00000051915 | 58,784,148 | Phosphodiesterase 5A, cgmp-specific, b |
| Contig2770 | ENSDARG00000043010 | 58,970,699 | Calcium/calmodulin-dependent protein kinase (cam kinase) II delta 1 |
| Contig2770 | ENSDARG00000043313 | 59,131,813 | Ankyrin 2, neuronal |
| Contig2770 | ENSDARG00000051920 | 59,387,629 | Neutral sphingomyelinase (N-smase) activation associated factor |
| Contig2770 | ENSDARG00000078703 | 59,431,589 | Aspartate beta-hydroxylase |
| Contig2770 | ENSDARG00000051926 | 59,975,152 | Pleiomorphic adenoma gene 1 |
| Contig2770 | ENSDARG00000010332 | 59,996,663 | Kinesin-like protein KIF20A |
| Contig2665 | ENSDARG00000077645 | 60,214,962 | G protein-coupled receptor 78 |
| Contig2665 | ENSDARG00000090754 | 60,226,310 | Uncharacterized protein |
| Contig2665 | ENSDARG00000035544 | 60,554,991 | Alanine-glyoxylate aminotransferase 2-like 1 |
| Contig1918 | ENSDARG00000030307 | 60,590,807 | Heat shock protein 12B |
| Contig1918 | ENSDARG00000007385 | 60,624,250 | Chaperonin containing TCP1, subunit 7 (eta) |
| Contig1918 | ENSDARG00000051955 | 60,872,755 | Breast cancer metastasis-suppressor 1 |
| Contig0067 | ENSDARG00000077162 | 61,530,628 | Leucine-rich repeat and WD repeat-containing protein |
| Contig0067 | ENSDARG00000079584 | 61,655,761 | Zgc:194930 |
| Contig0067 | ENSDARG00000059693 | 61,774,366 | ADAM metallopeptidase domain 33 |
| Contig0067 | ENSDARG00000068258 | 62,028,654 | Leucine-rich repeats and calponin homology (CH) domain containing 4 |
| Contig1705 | ENSDARG00000078437 | 62,531,495 | Uncharacterized protein |
| Contig1705 | ENSDARG00000042728 | 62,619,690 | Phospholipase A2-activating protein |
| Contig1705 | ENSDARG00000052091 | 62,723,096 | Recombination signal binding protein for immunoglobulin kappa J region b |
| Contig1705 | ENSDARG00000013842 | 62,891,233 | TBC1 domain family, member 19 |
| Contig1705 | ENSDARG00000001776 | 62,964,154 | Stromal interaction molecule 2 |
| Contig0067 | ENSDARG00000056389 | 64,296,863 | Arfgap with rhogap domain, ankyrin repeat and PH domain 2 |
| Contig2770 | ENSDARG00000041338 | 65,353,116 | Mitochondrial ribosomal protein L15 |
| Contig2665 | ENSDARG00000035838 | 65,684,911 | Oligosaccharyltransferase complex subunit |
| Contig1918 | ENSDARG00000051935 | 65,728,496 | Docking protein 1b |
| Contig1918 | ENSDARG00000079014 | 65,850,912 | Sema domain, immunoglobulin domain (Ig), transmembrane domain (TM) and short cytoplasmic domain, (semaphorin) 4F |
| Contig2570 | ENSDARG00000067829 | 73,134,952 | Peroxisome proliferator-activated receptor gamma, coactivator 1 alpha |
| Contig2570 | ENSDARG00000015392 | 73,835,483 | DEAH (Asp-Glu-Ala-His) box polypeptide 15 |
| Contig2498 | ENSDARG00000079906 | 74,456,967 | Calcium channel, voltage-dependent, beta 2a |
| Contig2570 | ENSDARG00000086141 | 75,095,954 | Leucine-rich repeat LGI family member 2 precursor |
| Contig2665 | ENSDARG00000026376 | 75,151,088 | Aconitase 1, soluble |
