# Supplementary material for: Comparative genomic analysis of catfish linkage group 8 reveals two homologous chromosomes in zebrafish and other teleosts with extensive inter-chromosomal rearrangements
Source: BMC Genomics. 2013 Jun 10;14:387. doi: 10.1186/1471-2164-14-387 (PMC3691659; doi:10.1186/1471-2164-14-387)
Supplement: Additional file 3 — Annotation of catfish genes mapped in one physical contig in LG8 with significant hits to both zebrafish chromosome 7 and chromosome 2. [file 1471-2164-14-387-S3.docx]

### S Table 3 - Annotation of catfish genes mapped in one physical map contig in LG8 with significant hits to both zebrafish chromosome 7 and chromosome 2.

| **BAC contig ID** | **Chr** | **Gene start** | **Gene ID** | **Description** |
| --- | --- | --- | --- | --- |
| Contig2577 | 2 | 5,749,025 | **ENSDARG00000058734** | Peroxiredoxin 1 |
| Contig2577 | 2 | 5,794,512 | ENSDARG00000076996 | Importin 13 |
| Contig2577 | 2 | 5,854,595 | ENSDARG00000078329 | REC8 homolog |
| Contig2577 | 2 | 5,903,803 | ENSDARG00000063223 | ADP-ribosylation factor-like 14 |
| Contig2577 | 2 | 7,845,116 | ENSDARG00000039272 | Plastin 1 (I isoform) |
| Contig2577 | 2 | 8,371,294 | ENSDARG00000058638 | Si:ch211-71m22.1 |
| Contig2577 | 2 | 8,433,634 | ENSDARG00000029248 | Far upstream element (FUSE) binding protein 1 |
| Contig2577 | 2 | 8,590,536 | ENSDARG00000077581 | Zinc finger, ZZ-type containing 3 |
| Contig2577 | 2 | 8,630,181 | ENSDARG00000012555 | Adenylate kinase 5 |
| Contig2577 | 2 | 9,114,487 | ENSDARG00000058473 | St6 |
| Contig2577 | 2 | 37,736,781 | ENSDARG00000053802 | Cerebellin 5 |
| Contig2577 | 7 | 4,261,604 | **ENSDARG00000022579** | Heterogeneous nuclear ribonucleoprotein |
| Contig2577 | 7 | 42,050,533 | **ENSDARG00000024092** | Limb region 1 homolog |
| Contig2577 | 7 | 42,111,456 | ENSDARG00000060035 | Ring finger protein 32 |
| Contig2577 | 7 | 43,738,977 | **ENSDARG00000010658** | Insulin induced gene 1 |
| Contig0123 | 2 | 30,178,657 | ENSDARG00000068483 | Neuropilin (NRP) and tolloid (TLL)-like 1 |
| Contig0123 | 2 | 30,204,678 | ENSDARG00000076066 | Membrane-associated ring finger (C3HC4) 6 |
| Contig0123 | 2 | 30,246,558 | ENSDARG00000015589 | Ankyrin repeat domain 33Bb |
| Contig0123 | 2 | 46,498,776 | ENSDARG00000076757 | EPH receptor B1 |
| Contig0123 | 7 | 43,876,308 | ENSDARG00000001241 | Poly-U binding splicing factor b |
| Contig0123 | 7 | 43,946,691 | ENSDARG00000000861 | Scribbled homolog |
| Contig0123 | 7 | 44,004,972 | ENSDARG00000031693 | Kinesin family member 9 |
| Contig0123 | 7 | 44,033,199 | ENSDARG00000009466 | Regulator of G-protein signalling 9 binding protein |
| Contig0123 | 7 | 44,070,147 | ENSDARG00000017242 | Family with sequence similarity 59, member A |
| Contig0123 | 7 | 44,078,350 | ENSDARG00000075083 | Protein kinase, DNA-activated, catalytic polypeptide |
| Contig0123 | 7 | 44,134,315 | ENSDARG00000052331 | ATP-binding cassette, sub-family F (GCN20), member 2 |
| Contig0123 | 7 | 44,221,134 | ENSDARG00000076662 | Nuclear transcription factor, X-box binding 1 |
| Contig2102 | 2 | 30,701,828 | ENSDARG00000061196 | Elastin microfibril interfacer 2a |
| Contig2102 | 2 | 31,098,754 | ENSDARG00000061140 | Collectin sub-family member 12 |
| Contig2102 | 2 | 38,073,355 | ENSDARG00000078253 | Proteasome (prosome, macropain) subunit, beta type, 11 |
| Contig2102 | 2 | 38,257,744 | ENSDARG00000077996 | Cadherin 24, type 2 |
| Contig2102 | 7 | 65,408,686 | ENSDARG00000073928 | Mannose receptor, C type 1a |
| Contig2102 | 7 | 75,222,916 | ENSDARG00000042894 | Thymidylate synthase |
| Contig2102 | 7 | 75,264,028 | ENSDARG00000088999 | Methyltransferase like 4 |

Gene IDs with same color-shadow means that the genes were mapped to the same BAC clone but with different BESs. For instance, ENSDARG00000076066 and ENSDARG00000009466 were mapped to the two different BESs of a BAC clone. Gene IDs bolded and with underlines indicate these genes were mapped to the same BAC contigs even based on the physical map with high stringency of p=10^-40^.
