# Supplementary material for: Comparative genomic analysis of catfish linkage group 8 reveals two homologous chromosomes in zebrafish and other teleosts with extensive inter-chromosomal rearrangements
Source: BMC Genomics. 2013 Jun 10;14:387. doi: 10.1186/1471-2164-14-387 (PMC3691659; doi:10.1186/1471-2164-14-387)
Supplement: Additional file 4 — Catfish genes mapped in LG8 with significant hits to Medaka chromosome 17. Microsyntenies are indicated by the same colored rows. [file 1471-2164-14-387-S4.docx]

**S Table 4. Catfish genes mapped in LG8 with significant hits to Medaka chromosome 17. Microsyntenies deteccted are indicated by the same colored rows.**

| **BAC contig ID** | **Gene ID** | **Gene Start** | **Description** |
| --- | --- | --- | --- |
| Contig2664 | ENSORLG00000002699 | 2,812,777 | Kin of irre like |
| Contig1016 | ENSORLG00000002717 | 2,914,969 | Potassium inwardly-rectifying channel, subfamily j, member 10 |
| Contig2664 | ENSORLG00000002786 | 3,053,349 | Poliovirus receptor-related 4 |
| Contig0672 | ENSORLG00000002857 | 3,096,064 | Coatomer protein complex, subunit alpha |
| Contig0672 | ENSORLG00000003084 | 3,405,958 | Apolipoprotein l domain containing 1 |
| Contig2577 | ENSORLG00000003144 | 3,542,417 | Uncharacterized protein |
| Contig2577 | ENSORLG00000003298 | 3,811,457 | Alpha 2,6 sialyltransferase iii |
| Contig2577 | ENSORLG00000003322 | 4,040,822 | Uncharacterized protein |
| Contig2577 | ENSORLG00000003342 | 4,150,262 | Zinc finger, zz-type containing 3 |
| Contig2577 | ENSORLG00000003540 | 4,218,782 | Far upstream element (fuse) binding protein 1 |
| Contig1723 | ENSORLG00000004365 | 5,261,686 | Biliverdin reductase a |
| Contig2727 | ENSORLG00000004486 | 5,416,549 | Uncharacterized protein |
| Contig2727 | ENSORLG00000005605 | 6,688,223 | Uncharacterized protein |
| Contig2535 | ENSORLG00000006195 | 8,630,282 | Uncharacterized protein |
| Contig2535 | ENSORLG00000006308 | 9,045,824 | Uncharacterized protein |
| Contig1724 | ENSORLG00000006337 | 9,103,021 | Zinc finger protein 622 |
| Contig0481 | ENSORLG00000006913 | 10,807,536 | Tetratricopeptide repeat domain 19 |
| Singleton | ENSORLG00000007419 | 12,640,609 | Laminin gamma-1 |
| Contig1723 | ENSORLG00000007769 | 12,965,363 | Xenotropic and polytropic retrovirus receptor 1 |
| Contig1723 | ENSORLG00000008404 | 14,039,683 | Receptor-interacting serine-threonine kinase 2 |
| Contig1723 | ENSORLG00000008793 | 14,632,505 | Coiled-coil domain containing 39 |
| Singleton | ENSORLG00000009374 | 15,210,765 | Dis3 mitotic control homolog (s. Cerevisiae)-like 2 |
| Contig0570 | ENSORLG00000009379 | 15,237,043 | G protein-coupled receptor 158 |
| Contig0570 | ENSORLG00000009513 | 15,441,983 | Abl-interactor 1 |
| Contig2732 | ENSORLG00000009804 | 15,926,038 | Complement component 8, alpha polypeptide |
| Contig2732 | ENSORLG00000009863 | 15,949,794 | Disabled homolog 1 (drosophila) |
| Contig2535 | ENSORLG00000009977 | 16,110,605 | Dynamin 3 |
| Contig2535 | ENSORLG00000010125 | 16,180,772 | Transcriptional adaptor 1 |
| Contig1723 | ENSORLG00000010871 | 16,851,867 | Uncharacterized protein |
| Contig1723 | ENSORLG00000010887 | 16,880,131 | Uncharacterized protein |
| Contig1723 | ENSORLG00000010916 | 17,098,406 | Regulator of g-protein signaling 18 |
| Contig1723 | ENSORLG00000011723 | 17,640,295 | Wd repeat domain 48 |
| Contig2102 | ENSORLG00000012385 | 18,216,932 | Cadherin 24, type 2 |
| Contig0779 | ENSORLG00000012525 | 18,843,972 | Solute carrier family 6 (neurotransmitter transporter, glycine), member 9 |
| Contig0123 | ENSORLG00000012760 | 19,883,584 | Ankyrin repeat domain 33b |
| Contig1676 | ENSORLG00000013978 | 21,777,845 | Solute carrier family 25 (pyrimidine nucleotide carrier ), member 36 |
| Contig1676 | ENSORLG00000014074 | 22,041,804 | Coatomer protein complex, subunit beta 2 (beta prime) |
| Contig0570 | ENSORLG00000014200 | 22,076,199 | Phosphate cytidylyltransferase 1, choline, alpha |
| Contig1724 | ENSORLG00000014565 | 22,378,360 | Uncharacterized protein |
| Contig0123 | ENSORLG00000015972 | 24,357,608 | Eph receptor b1 |
| Singleton | ENSORLG00000016137 | 24,963,047 | Patched 2 |
| Contig0850 | ENSORLG00000016212 | 25,090,152 | Polo-like kinase 3 |
| Contig2461 | ENSORLG00000016922 | 26,275,924 | Oxysterol binding protein-like 1a |
| Contig0034 | ENSORLG00000017110 | 26,916,284 | Uncharacterized protein |
| Contig0928 | ENSORLG00000017231 | 27,243,775 | Receptor (tnfrsf)-interacting serine-threonine kinase 1 |
| Contig0034 | ENSORLG00000017263 | 27,581,111 | Werner helicase interacting protein 1 |
| Contig0680 | ENSORLG00000017318 | 27,677,680 | Latrophilin 2 |
| Contig0034 | ENSORLG00000017500 | 28,277,254 | Suppression of tumorigenicity 18 |
| Contig2770 | ENSORLG00000017817 | 30,551,838 | Retinitis pigmentosa 1 |
| Contig2102 | ENSORLG00000017868 | 30,718,123 | Elastin microfibril interfacer 2 |
| Contig0672 | ENSORLG00000017963 | 31,409,687 | Protein kinase c, iota |
