# Supplementary material for: Comparative genomic analysis of catfish linkage group 8 reveals two homologous chromosomes in zebrafish and other teleosts with extensive inter-chromosomal rearrangements
Source: BMC Genomics. 2013 Jun 10;14:387. doi: 10.1186/1471-2164-14-387 (PMC3691659; doi:10.1186/1471-2164-14-387)
Supplement: Additional file 5 — Catfish genes mapped in LG8 with significant hits to medaka chromosome 18. Microsyntenies are indicated by the same colored rows. [file 1471-2164-14-387-S5.docx]

**S Table 5. Catfish genes mapped in LG8 with significant hits to medaka chromosome 18. Microsyntenies deteccted are indicated by the same colored rows.**

| **BAC contig ID** | **Gene ID** | **Gene Start** | **Description** |
| --- | --- | --- | --- |
| Contig1919 | ENSORLG00000003259 | 3,687,291 | Canopy 3 homolog |
| Contig0174 | ENSORLG00000003427 | 4,288,107 | Dysferlin, limb girdle muscular dystrophy 2b |
| Contig0174 | ENSORLG00000003465 | 4,620,695 | Cytochrome p450 26b1 |
| Contig1705 | ENSORLG00000003492 | 4,815,729 | Mus81 endonuclease homolog |
| Contig2813 | ENSORLG00000003518 | 4,863,576 | Uncharacterized protein |
| Contig2423 | ENSORLG00000003655 | 5,510,823 | Grb10 interacting gyf protein 1 |
| Contig1919 | ENSORLG00000003689 | 5,866,958 | Uncharacterized protein |
| Contig1919 | ENSORLG00000003766 | 6,107,791 | Dynein, axonemal, heavy chain 2 |
| Contig1919 | ENSORLG00000003789 | 6,297,156 | Lysine (k)-specific demethylase 6b |
| Contig1919 | ENSORLG00000003795 | 6,308,411 | Transmembrane protein 88 |
| Contig2534 | ENSORLG00000003985 | 6,413,231 | Uncharacterized protein |
| Contig2570 | ENSORLG00000004788 | 10,183,158 | Peroxisome proliferator-activated receptor gamma, coactivator 1 alpha |
| Contig2570 | ENSORLG00000004819 | 10,593,171 | Deah (asp-glu-ala-his) box polypeptide 15 |
| Contig2570 | ENSORLG00000004829 | 10,641,854 | Coiled-coil domain containing 149 |
| Contig2665 | ENSORLG00000004900 | 10,780,842 | Oligosaccharyltransferase complex subunit |
| Contig2665 | ENSORLG00000004922 | 10,784,789 | Alanine-glyoxylate aminotransferase 2-like 1 |
| Contig1705 | ENSORLG00000005124 | 11,240,257 | Phospholipase a2-activating protein |
| Contig1705 | ENSORLG00000005367 | 12,097,682 | Solute carrier family 3 (activators of dibasic and neutral amino acid transport), member 2 |
| Contig1705 | ENSORLG00000005383 | 12,106,367 | Sorting nexin 15 |
| Contig1705 | ENSORLG00000005386 | 12,128,335 | Uncharacterized protein |
| Contig1918 | ENSORLG00000005411 | 12,145,571 | Breast cancer metastasis suppressor 1 |
| Contig2120 | ENSORLG00000005511 | 12,427,464 | Uncharacterized protein |
| Contig2120 | ENSORLG00000005740 | 12,578,649 | Map/microtubule affinity-regulating kinase 2 |
| Contig2120 | ENSORLG00000005756 | 12,636,390 | Uncharacterized protein |
| Contig1918 | ENSORLG00000005819 | 12,642,775 | Chaperonin containing tcp1, subunit 7 (eta) |
| Contig1918 | ENSORLG00000005839 | 12,654,023 | Heat shock 70kd protein 12b |
| Contig1918 | ENSORLG00000005897 | 12,704,107 | Uncharacterized protein |
| Contig1918 | ENSORLG00000005915 | 12,867,411 | Sema domain, immunoglobulin domain (ig), transmembrane domain (tm) and short cytoplasmic domain, (semaphorin) 4f |
| Contig2664 | ENSORLG00000006000 | 13,958,447 | Uncharacterized protein |
| Contig2664 | ENSORLG00000006017 | 13,978,669 | Deltex homolog 4 |
| Contig2665 | ENSORLG00000006091 | 14,268,479 | G protein-coupled receptor 78 |
| Contig2577 | ENSORLG00000006122 | 14,341,121 | Phospholipid scramblase 3 |
| Contig2665 | ENSORLG00000006247 | 14,404,564 | Eukaryotic translation initiation factor 5a-like 1 |
| Contig1918 | ENSORLG00000006684 | 14,839,,661 | Solute carrier family 16, member 13 (monocarboxylic acid transporter 13) |
| Contig1919 | ENSORLG00000006715 | 14,851,485 | Arfgap with coiled-coil, ankyrin repeat and ph domains 1 |
| Contig1919 | ENSORLG00000006732 | 14,894,242 | Uncharacterized protein |
| Contig1919 | ENSORLG00000006827 | 15,037,668 | Uncharacterized protein |
| Contig1919 | ENSORLG00000006846 | 15,065,269 | Uncharacterized protein |
| Contig1919 | ENSORLG00000006869 | 15,096,001 | Netrin 3 |
| Contig0067 | ENSORLG00000006921 | 15,170,752 | Procollagen c-endopeptidase enhancer |
| Contig0067 | ENSORLG00000006929 | 15,189,752 | Period homolog 1 |
| Contig0067 | ENSORLG00000006947 | 15,306,653 | Kiaa1239 |
| Contig1705 | ENSORLG00000007017 | 17,512,322 | Mannosidase, alpha, class 2b, member 2 |
| Contig1705 | ENSORLG00000007164 | 17,726,147 | Claudin 15 |
| Contig1919 | ENSORLG00000007194 | 17,791,729 | Methyltransferase like 3 |
| Contig1705 | ENSORLG00000007262 | 17,847,398 | Oxidase (cytochrome c) assembly 1-like |
| Contig1705 | ENSORLG00000007279 | 17,880,453 | Uncharacterized protein |
| Contig0726 | ENSORLG00000007547 | 18,299,782 | Sushi, von willebrand factor type a, egf and pentraxin domain containing 1 |
| Contig0726 | ENSORLG00000007604 | 18,456,151 | Haus augmin-like complex, subunit 3 |
| Contig0839 | ENSORLG00000007633 | 18,596,088 | Wolf-hirschhorn syndrome candidate 2 |
| Singleton | ENSORLG00000007643 | 18,603,098 | Fatty acid amide hydrolase 2 [source:hgnc symbol;acc:26440] |
| Contig0726 | ENSORLG00000007721 | 18,684,821 | Phosphoinositide-3-kinase, class 3 |
| Contig0839 | ENSORLG00000007781 | 18,735,564 | Chromosome 20 open reading frame 27 |
| Contig0688 | ENSORLG00000007884 | 19,019,460 | Inturned planar cell polarity effector homolog |
| Contig0688 | ENSORLG00000007951 | 19,129,060 | Nitrilase 1 |
| Contig0688 | ENSORLG00000007974 | 19,163,388 | Uncharacterized protein |
| Contig0688 | ENSORLG00000008010 | 19,203,908 | Ras and rab interactor 1 |
| Contig0688 | ENSORLG00000008014 | 19,313,451 | Galactose-3-o-sulfotransferase 3 |
| Contig0688 | ENSORLG00000008041 | 19,411,462 | Copper chaperone for superoxide dismutase |
| Contig0688 | ENSORLG00000008092 | 19,502,453 | Uncharacterized protein |
| Contig0688 | ENSORLG00000008133 | 19,520,124 | Kinesin light chain 2 |
| Contig0688 | ENSORLG00000008177 | 19,545,294 | Phosphofurin acidic cluster sorting protein 1 |
| Contig1016 | ENSORLG00000008235 | 19,651,043 | Splicing factor 3b, subunit 2, 145kda |
| Contig1016 | ENSORLG00000008280 | 19,712,644 | Calpain 1, (mu/i) large subunit |
| Contig1258 | ENSORLG00000008366 | 20,271,807 | Integrin beta 1 binding protein (melusin) 2 |
| Contig1258 | ENSORLG00000008422 | 20,439,829 | Uncharacterized protein |
| Contig2813 | ENSORLG00000008493 | 21,136,300 | Cyclin b3 |
| Contig2813 | ENSORLG00000008504 | 21,174,160 | Uncharacterized protein |
| Contig2813 | ENSORLG00000008602 | 21,248,042 | Chloride channel, voltage-sensitive 5 |
| Contig1919 | ENSORLG00000009315 | 21,729,983 | Collagen, type iv, alpha 6 |
| Contig1919 | ENSORLG00000009512 | 21,933,264 | Guanylate cyclase olgc-r2 |
| Contig1919 | ENSORLG00000009559 | 22,002,837 | Rho gtpase activating protein 36 |
| Contig1919 | ENSORLG00000009563 | 22,056,155 | Nhs-like 2 |
| Contig1919 | ENSORLG00000009584 | 22,129,818 | Ribosomal protein s4x pseudogene 21 |
| Contig2120 | ENSORLG00000009606 | 22,154,121 | Histone deacetylase 8 |
| Contig2120 | ENSORLG00000009902 | 22,173,875 | Phosphorylase kinase, alpha 1 |
| Contig2214 | ENSORLG00000010064 | 22,468,704 | Uncharacterized protein |
| Contig2534 | ENSORLG00000010098 | 22,643,679 | Uncharacterized protein |
| Contig2577 | ENSORLG00000010157 | 22,878,723 | Uncharacterized protein |
| Contig1705 | ENSORLG00000010246 | 25,075,149 | Uncharacterized protein |
| Contig2770 | ENSORLG00000010255 | 25,104,480 | Uncharacterized protein |
| Contig2214 | ENSORLG00000010331 | 25,462,132 | Arachidonate 12-lipoxygenas |
| Contig1919 | ENSORLG00000010797 | 26,757,004 | Uncharacterized protein |
| Contig2214 | ENSORLG00000010894 | 26,898,056 | Uncharacterized protein |
| Contig2120 | ENSORLG00000011219 | 28,274,649 | Methyltransferase like 12 |
| Contig2664 | ENSORLG00000011324 | 28,336,162 | Nadh dehydrogenase (ubiquinone) fe-s protein 2, 49kda (nadh-coenzyme q reductase) |
| Contig2664 | ENSORLG00000011441 | 28,430,994 | Dedicator of cytokinesis 11 |
| Contig2813 | ENSORLG00000011571 | 28,532,695 | Fragile x mental retardation, autosomal homolog 2 |
| Contig2664 | ENSORLG00000011632 | 28,602,968 | Mitogen-activated protein kinase kinase kinase kinase 2 |
| Contig2664 | ENSORLG00000011682 | 28,650,883 | Reticulon 3 |
| Contig2664 | ENSORLG00000011690 | 28,687,143 | Uncharacterized protein |
| Contig2664 | ENSORLG00000011743 | 28,737,775 | Echinoderm microtubule associated protein like 3 |
| Contig2664 | ENSORLG00000011799 | 28,769,739 | Metastasis associated 1 family, member 2 |
| Contig2664 | ENSORLG00000011816 | 28,792,216 | Taf6-like rna polymerase ii, p300/cbp-associated factor (pcaf)-associated factor, 65kda |
| Contig2770 | ENSORLG00000011839 | 28,802,009 | Eh domain binding protein 1-like 1 |
| Contig2664 | ENSORLG00000012069 | 29,192,426 | Rna (guanine-9-) methyltransferase domain containing 2 |
| Contig2664 | ENSORLG00000012145 | 29,235,936 | Focadhesin |
| Contig2664 | ENSORLG00000012166 | 29,279,923 | Myeloid/lymphoid or mixed-lineage leukemia (trithorax homolog, drosophila); translocated to, 3 |
| Contig0067 | ENSORLG00000012217 | 29,344,534 | Leucine-rich repeats and calponin homology (ch) domain containing 4 |
| Contig0067 | ENSORLG00000012240 | 29,437,103 | Adam metallopeptidase domain 33 |
