# Supplementary material for: Comparative genomic analysis of catfish linkage group 8 reveals two homologous chromosomes in zebrafish and other teleosts with extensive inter-chromosomal rearrangements
Source: BMC Genomics. 2013 Jun 10;14:387. doi: 10.1186/1471-2164-14-387 (PMC3691659; doi:10.1186/1471-2164-14-387)
Supplement: Additional file 6 — Summary of conserved syntenic blocks between catfish LG8 and medaka chromosome 17. The number in parentheses mean the different snyteny within same physical contig. [file 1471-2164-14-387-S6.docx]

**S Table 6 -Summary of conserved syntenic blocks between catfish LG8 and medaka chromosome 17. The number** [**in parentheses**](app:ds:Within%20Parentheses) **mean the different snyteny within same physical contig.**

| **Syntenic blocks on medaka Chr17** | **Catfish BAC contigs** | **Number of genes** | **Spanning size**  **(kb)** |
| --- | --- | --- | --- |
| 1 | Contig0672 | 2 | 311 |
| 2 | Contig2577 | 5 | 684 |
| 3 | Contig2727 | 2 | 1,274 |
| 4 | Contig2535 ( 1 ) | 2 | 445 |
| 5 | Contig1723 ( 1 ) | 3 | 1,677 |
| 6 | Contig0570 | 2 | 221 |
| 7 | Contig2732 | 2 | 47 |
| 8 | Contig2535 ( 2 ) | 2 | 76 |
| 9 | Contig1723 ( 2 ) | 4 | 795 |
| 10 | Contig1676 | 2 | 269 |
| Total | 8 | 26 | 5,802 |
