# Supplementary material for: Comparative genomic analysis of catfish linkage group 8 reveals two homologous chromosomes in zebrafish and other teleosts with extensive inter-chromosomal rearrangements
Source: BMC Genomics. 2013 Jun 10;14:387. doi: 10.1186/1471-2164-14-387 (PMC3691659; doi:10.1186/1471-2164-14-387)
Supplement: Additional file 7 — Summary of conserved syntenic blocks between catfish LG8 and medaka chromosome 18. The number in parentheses mean the different snyteny within same physical contig. [file 1471-2164-14-387-S7.docx]

**S Table 7 -Summary of conserved syntenic blocks between catfish LG8 and medaka chromosome 18. The number** [**in parentheses**](app:ds:Within%20Parentheses) **mean the different snyteny within same physical contig.**

| **Syntenic block on medaka Chr18** | **Catfish BACcontigs** | **Number of genes** | **Spanning size**  **(kb)** |
| --- | --- | --- | --- |
| 1 | Contig0174 | 2 | 365 |
| 2 | Contig1919 (1) | 4 | 452 |
| 3 | Contig2570 | 3 | 466 |
| 4 | Contig2665 | 2 | 15 |
| 5 | Contig1705 (1) | 4 | 894 |
| 6 | Contig2120 (1) | 3 | 215 |
| 7 | Contig1918 | 4 | 239 |
| 8 | Contig2664 (1) | 2 | 34 |
| 9 | Contig1919 (2) | 5 | 258 |
| 10 | Contig0067 (1) | 3 | 148 |
| 11 | Contig1705 (2) | 2 | 218 |
| 12 | Contig1705 (3) | 2 | 39 |
| 13 | Contig0726 | 2 | 161 |
| 14 | Contig0688 | 9 | 565 |
| 15 | Contig1016 | 2 | 107 |
| 16 | Contig1258 | 2 | 169 |
| 17 | Contig2813 | 3 | 132 |
| 18 | Contig1919 (3) | 5 | 404 |
| 19 | Contig2120 (2) | 2 | 51 |
| 20 | Contig2664 (2) | 2 | 181 |
| 21 | Contig2664 (3) | 6 | 196 |
| 22 | Contig2664 (4) | 3 | 125 |
| 23 | Contig0067 (2) | 2 | 119 |
| Total | 14 | 74 | 5,553 |
