# Supplementary material for: Comparative genomic analysis of catfish linkage group 8 reveals two homologous chromosomes in zebrafish and other teleosts with extensive inter-chromosomal rearrangements
Source: BMC Genomics. 2013 Jun 10;14:387. doi: 10.1186/1471-2164-14-387 (PMC3691659; doi:10.1186/1471-2164-14-387)
Supplement: Additional file 8 — Catfish genes mapped in LG8 with significant hits to stickleback chromosome 3. Microsyntenies are indicated by the same colored rows. [file 1471-2164-14-387-S8.docx]

**S Table 8 -Catfish genes mapped in LG8 with significant hits to stickleback chromosome 3. Microsyntenies deteccted are indicated by the same colored rows.**

| **BAC Contig ID** | **Gene ID** | **Gene Start (bp)** | **Description** |
| --- | --- | --- | --- |
| Contig1723 | ENSGACG00000012625 | 175,029 | WD repeat domain 48 |
| Contig0928 | ENSGACG00000012991 | 469,854 | Receptor (TNFRSF)-interacting serine-threonine kinase 1 |
| Contig0034 | ENSGACG00000013161 | 694,549 | Uncharacterized protein |
| Contig2461 | ENSGACG00000013253 | 1,075,302 | Histamine receptor H4 |
| Contig2461 | ENSGACG00000013256 | 1,085,397 | Oxysterol binding protein-like 1A |
| Contig0850 | ENSGACG00000013777 | 1,970,047 | Polo-like kinase 3 |
| Singleton | ENSGACG00000013809 | 2,055,661 | Patched 2 |
| Contig2577 | ENSGACG00000013840 | 2,130,944 | ADP-ribosylation factor-like 14 |
| Contig1723 | ENSGACG00000013849 | 2,158,123 | Kiaa1614 |
| Contig1723 | ENSGACG00000013852 | 2,160,098 | Kiaa1614 |
| Contig0123 | ENSGACG00000013951 | 2,492,450 | EPH receptor B1 |
| Contig2102 | ENSGACG00000014245 | 3,057,406 | Cadherin 24, type 2 |
| Contig1676 | ENSGACG00000014253 | 3,157,734 | Leucine rich repeat containing 16B |
| Contig1676 | ENSGACG00000014265 | 3,229,059 | RAS (RAD and GEM)-like GTP binding 2 |
| Contig0779 | ENSGACG00000014462 | 4,456,398 | Solute carrier family 6 (neurotransmitter transporter, glycine), member 9 |
| Contig0123 | ENSGACG00000014515 | 4,951,552 | Neuropilin (NRP) and tolloid (TLL)-like 1 |
| Contig0123 | ENSGACG00000014533 | 4,984,417 | Ankyrin repeat domain 33B |
| Contig1723 | ENSGACG00000014536 | 5,002,434 | Xenotropic and polytropic retrovirus receptor 1 |
| Contig1723 | ENSGACG00000014566 | 5,088,854 | Biliverdin reductase A |
| Contig1724 | ENSGACG00000015386 | 7,784,960 | Uncharacterized protein |
| Contig0570 | ENSGACG00000015442 | 8,030,970 | Phosphate cytidylyltransferase 1, choline, alpha |
| Contig1676 | ENSGACG00000015455 | 8,059,592 | Coatomer protein complex, subunit beta 2 (beta prime) |
| Contig1676 | ENSGACG00000015468 | 8,127,912 | Calsyntenin 2 |
| Contig1676 | ENSGACG00000015473 | 8,228,521 | Solute carrier family 25 (pyrimidine nucleotide carrier ), member 36 |
| Contig1723 | ENSGACG00000016028 | 9,192,697 | Receptor-interacting serine-threonine kinase 2 |
| Contig1723 | ENSGACG00000016039 | 9,212,884 | Coiled-coil domain containing 39 |
| Contig1723 | ENSGACG00000016065 | 9,230,992 | UDP-glcnac:betagal beta-1,3-N-acetylglucosaminyltransferase 5 |
| Contig1723 | ENSGACG00000016122 | 9,445,520 | Regulator of G-protein signaling 18 |
| Contig1723 | ENSGACG00000016127 | 9,523,275 | Uncharacterized protein |
| Contig2577 | ENSGACG00000016172 | 9,656,798 | Peroxiredoxin 1 |
| Contig2535 | ENSGACG00000016268 | 9,917,839 | Transcriptional adaptor 1 |
| Contig2535 | ENSGACG00000016290 | 9,947,686 | Dynamin 3 |
| Contig2732 | ENSGACG00000016314 | 10,029,934 | Disabled homolog 1 |
| Contig2732 | ENSGACG00000016338 | 10,062,736 | Complement component 8, alpha polypeptide |
| Contig0570 | ENSGACG00000016406 | 10,329,729 | Abl-interactor 1 |
| Singleton | ENSGACG00000016435 | 10,417,812 | DIS3 mitotic control homolog (S. Cerevisiae)-like 2 |
| Contig0034 | ENSGACG00000016469 | 10,620,517 | Myosin light chain kinase family, member 4 |
| Contig2577 | ENSGACG00000016529 | 10,691,525 | St6 |
| Contig2577 | ENSGACG00000016538 | 10,789,219 | Adenylate kinase 5 |
| Contig2577 | ENSGACG00000016546 | 10,831,750 | Zinc finger, ZZ-type containing 3 |
| Contig2577 | ENSGACG00000016563 | 10,874,901 | Far upstream element (FUSE) binding protein 1 |
| Contig0672 | ENSGACG00000016956 | 12,445,195 | Protein kinase C, iota |
| Contig2102 | ENSGACG00000017058 | 12,763,399 | Collectin sub-family member 12 |
| Contig2102 | ENSGACG00000017089 | 12,893,878 | Elastin microfibril interfacer 2 |
| Contig2535 | ENSGACG00000017559 | 14,324,861 | Uncharacterized protein |
| Contig2664 | ENSGACG00000017724 | 15,845,338 | Poliovirus receptor-related 4 |
| Contig0672 | ENSGACG00000017751 | 15,879,522 | Coatomer protein complex, subunit alpha |
| Contig2577 | ENSGACG00000017772 | 15,930,985 | Uncharacterized protein |
| Contig0672 | ENSGACG00000017779 | 15,961,842 | Apolipoprotein L domain containing 1 |
| Contig0034 | ENSGACG00000017821 | 16,064,588 | Werner helicase interacting protein 1 |
| Contig0680 | ENSGACG00000017847 | 16,124,121 | Latrophilin 2 |
| Contig0034 | ENSGACG00000017947 | 16,479,364 | Suppression of tumorigenicity 18 (breast carcinoma) \ |
| Contig1724 | ENSGACG00000018006 | 16,787,629 | Zinc finger protein 622 |
