# Supplementary material for: Comparative genomic analysis of catfish linkage group 8 reveals two homologous chromosomes in zebrafish and other teleosts with extensive inter-chromosomal rearrangements
Source: BMC Genomics. 2013 Jun 10;14:387. doi: 10.1186/1471-2164-14-387 (PMC3691659; doi:10.1186/1471-2164-14-387)
Supplement: Additional file 9 — Catfish genes mapped in LG8 with significant hits to stickleback chromosome 7. Microsyntenies are indicated by the same colored rows. [file 1471-2164-14-387-S9.docx]

**S Table 9 -Catfish genes mapped in LG8 with significant hits to stickleback chromosome 7. Microsyntenies deteccted are indicated by the same colored rows.**

| **BAC contig ID** | **Ensembl Gene ID** | **Gene Start (bp)** | **Description** |
| --- | --- | --- | --- |
| Contig1919 | ENSGACG00000018704 | 590,628 | Guanylate cyclase 2f, retinal |
| Contig1919 | ENSGACG00000018720 | 697,432 | Ribosomal protein s4x pseudogene 21 |
| Contig2120 | ENSGACG00000018737 | 715,348 | Histone deacetylase 8 |
| Contig2120 | ENSGACG00000018743 | 727,279 | Phosphorylase kinase, alpha 1 (muscle) |
| Contig2813 | ENSGACG00000018753 | 787,500 | Chloride channel, voltage-sensitive 5 |
| Contig1705 | ENSGACG00000018793 | 1,113,582 | Mus81 endonuclease homolog |
| Contig0174 | ENSGACG00000018809 | 1,211,620 | Cytochrome p450, family 26, subfamily b, polypeptide 1 |
| Contig0174 | ENSGACG00000018813 | 1,289,872 | Dysferlin, limb girdle muscular dystrophy 2b |
| Contig2214 | ENSGACG00000018922 | 1,723,958 | Uncharacterized protein |
| Contig0928 | ENSGACG00000018984 | 2,209,135 | Uncharacterized protein |
| Contig1016 | ENSGACG00000018986 | 2,242,556 | Potassium inwardly-rectifying channel, subfamily j, member 10 |
| Contig1016 | ENSGACG00000018991 | 2,259,206 | Calpain 1, (mu/i) large subunit |
| Contig1016 | ENSGACG00000019012 | 2,271,611 | Splicing factor 3b, subunit 2, 145kda |
| Contig0688 | ENSGACG00000019019 | 2,280,760 | Phosphofurin acidic cluster sorting protein 1 |
| Contig0688 | ENSGACG00000019021 | 2,303,933 | Kinesin light chain 2 |
| Contig0688 | ENSGACG00000019029 | 2,338,333 | Copper chaperone for superoxide dismutase |
| Contig0688 | ENSGACG00000019030 | 2,346,844 | Galactose-3-o-sulfotransferase 3 |
| Contig0688 | ENSGACG00000019038 | 2,403,544 | Uncharacterized protein |
| Contig0688 | ENSGACG00000019044 | 2,420,217 | Nitrilase 1 |
| Contig1016 | ENSGACG00000019055 | 2,454,404 | Uncharacterized protein |
| Contig0688 | ENSGACG00000019072 | 2,483,542 | Inturned planar cell polarity effector homolog |
| Contig0839 | ENSGACG00000019096 | 2,613,060 | Chromosome 20 open reading frame 27 |
| Contig0726 | ENSGACG00000019111 | 2,686,302 | Phosphoinositide-3-kinase, class 3 |
| Singleton | ENSGACG00000019119 | 2,726,309 | Fatty acid amide hydrolase 2 |
| Contig0839 | ENSGACG00000019124 | 2,734,092 | Wolf-hirschhorn syndrome candidate 2 |
| Contig0726 | ENSGACG00000019137 | 2,804,573 | Haus augmin-like complex, subunit 3 |
| Contig0726 | ENSGACG00000019152 | 2,816,084 | Sushi, von willebrand factor type a, egf and pentraxin domain containing 1 |
| Contig1705 | ENSGACG00000019211 | 3,034,373 | Oxidase (cytochrome c) assembly 1-like |
| Contig1919 | ENSGACG00000019244 | 3,063,289 | Methyltransferase like 3 |
| Contig1705 | ENSGACG00000019261 | 3,092,201 | Claudin 15 |
| Contig1705 | ENSGACG00000019279 | 3,138,404 | Uncharacterized protein |
| Contig1705 | ENSGACG00000019289 | 3,162,385 | Mannosidase, alpha, class 2b, member 2 |
| Contig1705 | ENSGACG00000019295 | 3,222,487 | Tbc1 domain family, member 19 |
| Contig1705 | ENSGACG00000019297 | 3,248,713 | Stromal interaction molecule 2 |
| Contig0067 | ENSGACG00000019301 | 3,862,698 | Death domain containing 1 |
| Contig0067 | ENSGACG00000019305 | 3,943,127 | Kiaa1239 |
| Contig0067 | ENSGACG00000019308 | 3,993,749 | Period homolog 1 |
| Contig0067 | ENSGACG00000019312 | 4,003,245 | Procollagen c-endopeptidase enhancer |
| Contig1919 | ENSGACG00000019316 | 4,025,500 | Canopy 4 homolog |
| Contig1919 | ENSGACG00000019318 | 4,039,346 | Netrin 3 |
| Contig1919 | ENSGACG00000019319 | 4,050,458 | Uncharacterized protein |
| Contig1919 | ENSGACG00000019328 | 4,092,922 | Uncharacterized protein |
| Contig1919 | ENSGACG00000019339 | 4,179,253 | Arfgap with coiled-coil, ankyrin repeat and ph domains 1 |
| Contig1918 | ENSGACG00000019347 | 4,193,229 | Solute carrier family 16, member 13 |
| Contig2665 | ENSGACG00000019377 | 4,319,824 | Uncharacterized protein |
| Contig2665 | ENSGACG00000019411 | 4,413,660 | G protein-coupled receptor 78 |
| Contig2664 | ENSGACG00000019427 | 4,558,350 | Deltex homolog 4 |
| Contig2664 | ENSGACG00000019429 | 4,572,958 | Uncharacterized protein |
| Contig2570 | ENSGACG00000019546 | 6,333,047 | Peroxisome proliferator-activated receptor gamma, coactivator 1 alpha |
| Contig2665 | ENSGACG00000019601 | 6,666,220 | Oligosaccharyltransferase complex subunit |
| Contig2665 | ENSGACG00000019605 | 6,668,657 | Alanine-glyoxylate aminotransferase 2-like 1 |
| Contig1705 | ENSGACG00000019658 | 6,983,627 | Phospholipase a2-activating protein |
| Contig1705 | ENSGACG00000019745 | 7,463,600 | Solute carrier family 3 (activators of dibasic and neutral amino acid transport), member 2 |
| Contig1705 | ENSGACG00000019747 | 7,474,994 | Sorting nexin 15 |
| Contig1918 | ENSGACG00000019753 | 7,500,063 | Breast cancer metastasis suppressor 1 |
| Contig2120 | ENSGACG00000019790 | 7,658,493 | Uncharacterized protein |
| Contig2120 | ENSGACG00000019840 | 7,786,266 | Map/microtubule affinity-regulating kinase 2 |
| Contig2120 | ENSGACG00000019844 | 7,825,878 | Uncharacterized protein |
| Contig1918 | ENSGACG00000019847 | 7,831,193 | Chaperonin containing tcp1, subunit 7 (eta) |
| Contig1918 | ENSGACG00000019861 | 7,838,207 | Heat shock 70kd protein 12b |
| Contig1918 | ENSGACG00000019876 | 7,873,702 | Uncharacterized protein |
| Contig1918 | ENSGACG00000019881 | 7,969,174 | Sema domain, immunoglobulin domain (ig), transmembrane domain (tm) and short cytoplasmic domain, (semaphorin) 4f |
| Contig1919 | ENSGACG00000019919 | 8,149,984 | Transmembrane protein 88 |
| Contig1919 | ENSGACG00000019920 | 8,160,493 | Lysine (k)-specific demethylase 6b |
| Contig2423 | ENSGACG00000019925 | 8,583,416 | Grb10 interacting gyf protein 1 |
| Contig2813 | ENSGACG00000019954 | 9,000,505 | Uncharacterized protein |
| Contig2813 | ENSGACG00000019983 | 9,259,652 | Fragile x mental retardation, autosomal homolog 2 |
| Contig2664 | ENSGACG00000020006 | 9,370,306 | Nadh dehydrogenase (ubiquinone) fe-s protein 2, 49kda (nadh-coenzyme q reductase) |
| Contig2120 | ENSGACG00000020022 | 9,404,415 | Methyltransferase like 12 |
| Contig2535 | ENSGACG00000020218 | 14,226,053 | Uncharacterized protein |
| Contig1422 | ENSGACG00000020322 | 16,979,133 | Neuroligin 2 |
| Contig2664 | ENSGACG00000020337 | 17,198,387 | Chromosome x open reading frame 57 |
| Contig0174 | ENSGACG00000020375 | 17,690,575 | Myotubularin related protein 2 |
| Contig1919 | ENSGACG00000020399 | 17,978,322 | Uncharacterized protein |
| Contig2577 | ENSGACG00000020510 | 19,331,992 | Uncharacterized protein |
| Contig1258 | ENSGACG00000020588 | 20,741,461 | Cysteine and histidine-rich domain (chord) containing 1 |
| Contig2665 | ENSGACG00000020658 | 22,028,152 | Uncharacterized protein |
