# Supplementary material for: Comparative genomic analysis of catfish linkage group 8 reveals two homologous chromosomes in zebrafish and other teleosts with extensive inter-chromosomal rearrangements
Source: BMC Genomics. 2013 Jun 10;14:387. doi: 10.1186/1471-2164-14-387 (PMC3691659; doi:10.1186/1471-2164-14-387)
Supplement: Additional file 10 — Summary of conserved syntenic blocks between catfish LG8 and stickleback chromosome 3. The number in parentheses mean the different snyteny within same physical contig. [file 1471-2164-14-387-S10.docx]

**S Table 10 -Summary of conserved syntenic blocks between catfish LG8 and stickleback chromosome 3. The number** [**in parentheses**](app:ds:Within%20Parentheses) **mean the different snyteny within same physical contig.**

| **Syntenic block on stickleback Chr3** | **Catfish physical contigs** | **Number of genes** | **Spanning size**  **(kb)** |
| --- | --- | --- | --- |
| 1 | Contig2461 | 2 | 25 |
| 2 | Contig1723 | 2 | 5 |
| 3 | Contig1676 | 2 | 98 |
| 4 | Contig0123 | 2 | 39 |
| 5 | Contig1723 (1) | 2 | 91 |
| 6 | Contig1676 | 3 | 182 |
| 7 | Contig1723 (2) | 5 | 332 |
| 8 | Contig2535 | 2 | 46 |
| 9 | Contig2732 | 2 | 39 |
| 10 | Contig2577 | 4 | 190 |
| 11 | Contig2102 | 2 | 144 |
| Total | 10 | 28 | 1,191 |
