# Supplementary material for: Comparative genomic analysis of catfish linkage group 8 reveals two homologous chromosomes in zebrafish and other teleosts with extensive inter-chromosomal rearrangements
Source: BMC Genomics. 2013 Jun 10;14:387. doi: 10.1186/1471-2164-14-387 (PMC3691659; doi:10.1186/1471-2164-14-387)
Supplement: Additional file 11 — Summary of conserved syntenic blocks between catfish LG8 and stickleback chromosome 7. The number in parentheses mean the different snyteny within same physical contig. [file 1471-2164-14-387-S11.docx]

**S Table 11 -Summary of conserved syntenic blocks between catfish LG8 and stickleback chromosome 7. The number** [**in parentheses**](app:ds:Within%20Parentheses) **mean the different snyteny within same physical contig.**

| **Syntenic block on stickleback Chr7** | **Catfish physical contigs** | **Number of genes** | **Spanning size**  **(kb)** |
| --- | --- | --- | --- |
| 1 | Contig1919 (1) | 2 | 112 |
| 2 | Contig2120 (1) | 2 | 23 |
| 3 | Contig0174 | 2 | 112 |
| 4 | Contig1016 | 3 | 35 |
| 5 | Contig0688 | 6 | 141 |
| 6 | Contig0726 | 2 | 71 |
| 7 | Contig1705 (1) | 5 | 165 |
| 8 | Contig0067 | 4 | 146 |
| 9 | Contig1919 (2) | 5 | 167 |
| 10 | Contig2665 (1) | 2 | 96 |
| 11 | Contig2664 | 2 | 18 |
| 12 | Contig2665 (2) | 2 | 10 |
| 13 | Contig1705 (2) | 3 | 495 |
| 14 | Contig2120 (2) | 3 | 171 |
| 15 | Contig1918 | 4 | 143 |
| 16 | Contig1919 (3) | 2 | 14 |
| 17 | Contig2813 | 2 | 271 |
| **Total** | 12 | 51 | 2,190 |
