# Supplementary material for: Comparative genomic analysis of catfish linkage group 8 reveals two homologous chromosomes in zebrafish and other teleosts with extensive inter-chromosomal rearrangements
Source: BMC Genomics. 2013 Jun 10;14:387. doi: 10.1186/1471-2164-14-387 (PMC3691659; doi:10.1186/1471-2164-14-387)
Supplement: Additional file 12 — Catfish genes mapped in LG8 with significant hits to green-spotted pufferfish chromosome 15. Microsyntenies are indicated by the same colored rows. [file 1471-2164-14-387-S12.docx]

**S Table 12. Catfish genes mapped in LG8 with significant hits to green-spotted pufferfish chromosome 15. Microsyntenies deteccted are indicated by the same colored rows.**

| **BAC contig ID** | **Gene ID** | **Gene Start** | **Description** |
| --- | --- | --- | --- |
| Contig1724 | ENSTNIG00000008000 | 660,686 | Zinc finger protein 622 |
| Contig0779 | ENSTNIG00000007959 | 1,877,843 | Solute carrier family 6 (neurotransmitter transporter, glycine), member 9 |
| Contig1676 | ENSTNIG00000007951 | 2,012,757 | Leucine rich repeat containing 16b |
| Contig2102 | ENSTNIG00000007950 | 2,056,413 | Cadherin 24, type 2 |
| Contig1723 | ENSTNIG00000007917 | 2,407,412 | Wd repeat domain 48 |
| Singleton | ENSTNIG00000007891 | 2,645,826 | Dis3 mitotic control homolog (s. Cerevisiae)-like 2 |
| Contig0570 | ENSTNIG00000007890 | 2,653,869 | G protein-coupled receptor 158 |
| Contig0570 | ENSTNIG00000007886 | 2,693,759 | Abl-interactor 1 |
| Contig2732 | ENSTNIG00000007872 | 2,843,172 | Phosphatidic acid phosphatase type 2b |
| Contig2732 | ENSTNIG00000007871 | 2,855,975 | Complement component 8, alpha polypeptide |
| Contig2732 | ENSTNIG00000007869 | 2,864,472 | Disabled homolog 1 |
| Contig2535 | ENSTNIG00000007867 | 2,930,214 | Dynamin 3 |
| Contig2535 | ENSTNIG00000007862 | 2,965,906 | Transcriptional adaptor 1 |
| Contig0672 | ENSTNIG00000007850 | 3,067,663 | Low density lipoprotein receptor-related protein 8, apolipoprotein e receptor |
| Contig2535 | ENSTNIG00000007836 | 3,203,185 | Carboxypeptidase n, polypeptide 2 |
| Contig1723 | ENSTNIG00000007826 | 3,284,135 | Receptor-interacting serine-threonine kinase 2 |
| Contig1723 | ENSTNIG00000007824 | 3,295,637 | Coiled-coil domain containing 39 |
| Contig1723 | ENSTNIG00000007820 | 3,,310,442 | Udp-glcnac:betagal beta-1,3-n-acetylglucosaminyltransferase 5 |
| Contig1723 | ENSTNIG00000006932 | 3,448,076 | Regulator of g-protein signaling 18 |
| Contig1723 | ENSTNIG00000006935 | 3,503,873 | Uncharacterized protein |
| Contig1723 | ENSTNIG00000006936 | 3,509,376 | Uncharacterized protein |
| Contig2577 | ENSTNIG00000006948 | 3,593,873 | Chromosome 15 scaf14367, whole genome shotgun sequence.natural killer cell enhancement factor |
| Contig1919 | ENSTNIG00000006976 | 3,829,317 | Pif1 5'-to-3' dna helicase homolog |
| Contig1676 | ENSTNIG00000011083 | 4,173,302 | Chromosome 15 scaf14667, whole genome shotgun sequence. |
| Contig1676 | ENSTNIG00000011085 | 4,218,121 | Calsyntenin 2 |
| Contig1676 | ENSTNIG00000011087 | 4,281,853 | Coatomer protein complex, subunit beta 2 (beta prime) |
| Contig0570 | ENSTNIG00000011090 | 4,305,540 | Phosphate cytidylyltransferase 1, choline, alpha |
| Contig1724 | ENSTNIG00000006035 | 4,441,780 | Uncharacterized protein |
| Contig0123 | ENSTNIG00000015276 | 5,494,539 | Eph receptor b1 |
| Contig1723 | ENSTNIG00000015268 | 5,767,383 | Kiaa1614 |
| Contig0850 | ENSTNIG00000015250 | 6,014,824 | Polo-like kinase 3 |
| Singleton | ENSTNIG00000015248 | 6,066,562 | Patched 2 |
| Contig2577 | ENSTNIG00000015242 | 6,151,077 | Adp-ribosylation factor-like 14 |
| Contig2461 | ENSTNIG00000015223 | 6,649,732 | Oxysterol binding protein-like 1a |
| Contig2461 | ENSTNIG00000015222 | 6,666,028 | Chromosome 15 scaf14992, whole genome shotgun sequence. |
