# Supplementary material for: Comparative genomic analysis of catfish linkage group 8 reveals two homologous chromosomes in zebrafish and other teleosts with extensive inter-chromosomal rearrangements
Source: BMC Genomics. 2013 Jun 10;14:387. doi: 10.1186/1471-2164-14-387 (PMC3691659; doi:10.1186/1471-2164-14-387)
Supplement: Additional file 13 — Catfish genes mapped in LG8 with significant hits to green-spotted pufferfish chromosome 20 and chromosome 6. Microsyntenies are indicated by the same colored rows. [file 1471-2164-14-387-S13.docx]

**S Table 13. Catfish genes mapped in LG8 with significant hits to green-spotted pufferfish chromosome 20 and chromosome 6. Microsyntenies deteccted are indicated by the same colored rows.**

| **BAC contig ID** | **Chr** | **Gene ID** | **Gene Start** | **Description** |
| --- | --- | --- | --- | --- |
| Contig2665 | 20 | ENSTNIG00000010140 | 596,631 | Eukaryotic translation initiation factor 5a-like 1 |
| Contig2665 | 20 | ENSTNIG00000010147 | 643,973 | G protein-coupled receptor 78 |
| Contig2665 | 20 | ENSTNIG00000010148 | 649,116 | Uncharacterized protein |
| Contig1918 | 20 | ENSTNIG00000010152 | 683,492 | Solute carrier family 16, member 13 |
| Contig1919 | 20 | ENSTNIG00000010153 | 686,821 | Arfgap with coiled-coil, ankyrin repeat and ph domains 1 |
| Contig1919 | 20 | ENSTNIG00000002043 | 704,438 | Uncharacterized protein |
| Contig1919 | 20 | ENSTNIG00000010159 | 759,939 | Uncharacterized protein |
| Contig1919 | 20 | ENSTNIG00000010160 | 768,299 | Netrin 3 |
| Contig1919 | 20 | ENSTNIG00000006854 | 785,584 | Canopy 4 homolog |
| Contig0067 | 20 | ENSTNIG00000007199 | 804,053 | Procollagen c-endopeptidase enhancer |
| Contig0067 | 20 | ENSTNIG00000007198 | 810,004 | Period homolog 1 |
| Contig0067 | 20 | ENSTNIG00000007196 | 848,495 | Kiaa1239 |
| Contig1705 | 20 | ENSTNIG00000012991 | 1,272,517 | Stromal interaction molecule 2 |
| Contig1705 | 20 | ENSTNIG00000012992 | 1,284,982 | Tbc1 domain family, member 19 |
| Contig1705 | 20 | ENSTNIG00000012994 | 1,302,043 | Recombination signal binding protein for immunoglobulin kappa j region |
| Contig1705 | 20 | ENSTNIG00000012995 | 1,338,623 | Mannosidase, alpha, class 2b, member 2 |
| Contig1705 | 20 | ENSTNIG00000012997 | 1,360,053 | Uncharacterized protein |
| Contig1705 | 20 | ENSTNIG00000013001 | 1,401,666 | Claudin 15 |
| Contig1919 | 20 | ENSTNIG00000013005 | 1,418,791 | Methyltransferase like 3 |
| Contig1705 | 20 | ENSTNIG00000013010 | 1,455,282 | Oxidase (cytochrome c) assembly 1-like |
| Contig1705 | 20 | ENSTNIG00000013012 | 1,484,096 | Uncharacterized protein |
| Contig1918 | 20 | ENSTNIG00000013014 | 1,497,444 | Breast cancer metastasis suppressor 1 |
| Contig1705 | 20 | ENSTNIG00000013016 | 1,506,296 | Uncharacterized protein |
| Contig1705 | 20 | ENSTNIG00000013017 | 1,541,157 | Sorting nexin 15 |
| Contig1705 | 20 | ENSTNIG00000013018 | 1,552,153 | Solute carrier family 3 (activators of dibasic and neutral amino acid transport), member 2 |
| Contig0002 | 6 | ENSTNIG00000008150 | 2,190,656 | Shank-associated rh domain interactor |
| Contig0123 | 6 | ENSTNIG00000008163 | 2,355,467 | Membrane-associated ring finger (c3hc4) 6, e3 ubiquitin protein ligase |
| Contig2577 | 6 | ENSTNIG00000012760 | 4,447,267 | Phospholipid scramblase family, member 5 |
|  | 6 | ENSTNIG00000012775 | 4,841,118 | 5-hydroxytryptamine (serotonin) receptor 5a, g protein-coupled |
| Contig2577 | 6 | ENSTNIG00000012776 | 4,862,114 | Insulin induced gene 1 |
| Contig2577 | 6 | ENSTNIG00000012781 | 4,959,966 | Ring finger protein 32 |
| Contig2577 | 6 | ENSTNIG00000012782 | 4,967,047 | Limb region 1 homolog |
| Contig2534 | 6 | ENSTNIG00000012783 | 4,988,546 | Nucleolar protein with mif4g domain 1 |
| Contig2498 | 6 | ENSTNIG00000005846 | 5,129,609 | Chromosome 10 open reading frame 112 |
| Contig2498 | 6 | ENSTNIG00000017111 | 5,215,025 | Calcium channel, voltage-dependent, beta 2 |
| Contig2102 | 6 | ENSTNIG00000017113 | 5,258,755 | Uncharacterized protein |
| Contig2102 | 6 | ENSTNIG00000017118 | 5,388,050 | Methyltransferase like 4 |
| Contig0123 | 6 | ENSTNIG00000017143 | 5,743,775 | Atp-binding cassette, sub-family f (gcn20), member 2 |
| Contig0481 | 6 | ENSTNIG00000017145 | 5,765,728 | Solute carrier family 4, anion exchanger, member 2 (erythrocyte membrane protein band 3-like 1) |
| Contig0481 | 6 | ENSTNIG00000013676 | 5,814,775 | Nuclear receptor binding protein 2 |
| Contig0123 | 6 | ENSTNIG00000013675 | 5,820,049 | Poly-u binding splicing factor 60kda |
| Contig0123 | 6 | ENSTNIG00000013674 | 5,839,069 | Uncharacterized protein |
