# Supplementary material for: Comparative genomic analysis of catfish linkage group 8 reveals two homologous chromosomes in zebrafish and other teleosts with extensive inter-chromosomal rearrangements
Source: BMC Genomics. 2013 Jun 10;14:387. doi: 10.1186/1471-2164-14-387 (PMC3691659; doi:10.1186/1471-2164-14-387)
Supplement: Additional file 14 — Summary of conserved syntenic blocks between catfish LG8 and green-spotted pufferfish chromosome 15. The number in parentheses mean the different snyteny within same physical contig. [file 1471-2164-14-387-S14.docx]

**S Table 13 -Summary of conserved syntenic blocks between catfish LG8 and green-spotted pufferfish chromosome 15. The number** [**in parentheses**](app:ds:Within%20Parentheses) **mean the different snyteny within same physical contig.**

| **Syntenic block on Tetraondon Chr15** | **Catfish BAC contigs** | **Number of genes** | **Spanning size**  **(kb)** |
| --- | --- | --- | --- |
| 1 | Contig0570 | 2 | 50 |
| 2 | Contig2732 (1) | 3 | 37 |
| 3 | Contig2535 | 2 | 38 |
| 4 | Contig1723 (2) | 6 | 231 |
| 5 | Contig1676 | 3 | 114 |
| 6 | Contig2461 | 2 | 19 |
| **Total** | 5 | 18 | 488 |
