# Supplementary material for: Comparative genomic analysis of catfish linkage group 8 reveals two homologous chromosomes in zebrafish and other teleosts with extensive inter-chromosomal rearrangements
Source: BMC Genomics. 2013 Jun 10;14:387. doi: 10.1186/1471-2164-14-387 (PMC3691659; doi:10.1186/1471-2164-14-387)
Supplement: Additional file 15 — Summary of conserved syntenic blocks between catfish LG8 and green-spotted pufferfish chromosome 20 and chromosome 6. The number in parentheses mean the different snyteny within same physical contig. [file 1471-2164-14-387-S15.docx]

**S Table 15 -Summary of conserved syntenic blocks between catfish LG8 and green-spotted pufferfish chromosome 20 and chromosome 6. The number** [**in parentheses**](app:ds:Within%20Parentheses) **mean the different snyteny within same physical contig.**

| **Chromosome** | **Syntenic block** | **Catfish BAC contigs** | **Number of genes** | **Spanning size**  **(kb)** |
| --- | --- | --- | --- | --- |
| **Tetraondon**  **Chr 20** | 1 | Contig2665 | 3 | 55 |
|  | 2 | Contig1919 | 5 | 101 |
|  | 3 | Contig0067 | 3 | 58 |
|  | 4 | Contig1705 (1) | 6 | 131 |
|  | 5 | Contig1705 (2) | 2 | 30 |
|  | 6 | Contig1705 (3) | 3 | 49 |
|  | **Total** | **4** | **22** | **424** |
| **Tetraondon**  **Chr 6** | 1 | Contig2577 | 4 | 541 |
|  | 2 | Contig2498 | 2 | 97 |
|  | 3 | Contig2102 | 2 | 134 |
|  | 4 | Contig0481 | 2 | 51 |
|  | 5 | Contig0123 | 2 | 37 |
|  | **Total** | **5** | **12** | **860** |
