# Supplementary material for: Comparative genomic analysis of catfish linkage group 8 reveals two homologous chromosomes in zebrafish and other teleosts with extensive inter-chromosomal rearrangements
Source: BMC Genomics. 2013 Jun 10;14:387. doi: 10.1186/1471-2164-14-387 (PMC3691659; doi:10.1186/1471-2164-14-387)
Supplement: Additional file 16 — Comparative map between catfish LG8 and medaka chromosome 17 and chromosome 18. [file 1471-2164-14-387-S16.pdf]

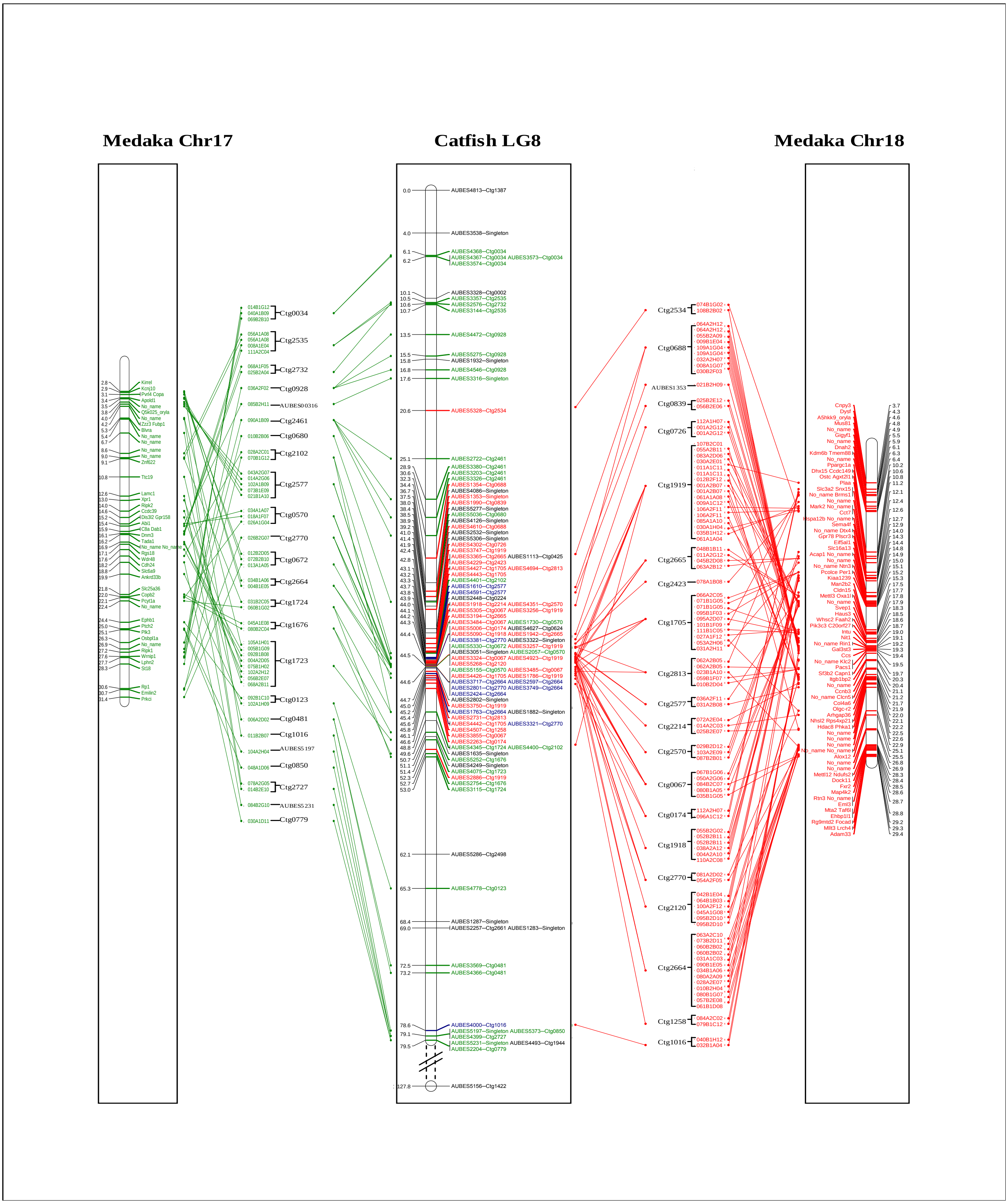
Medaka Chr17
Catfish LG8
Medaka Chr18
