# Supplementary material for: Comparative genomic analysis of catfish linkage group 8 reveals two homologous chromosomes in zebrafish and other teleosts with extensive inter-chromosomal rearrangements
Source: BMC Genomics. 2013 Jun 10;14:387. doi: 10.1186/1471-2164-14-387 (PMC3691659; doi:10.1186/1471-2164-14-387)
Supplement: Additional file 17 — Comparative map between catfish LG8 and stickleback chromosome 3 and chromosome 7. [file 1471-2164-14-387-S17.pdf]

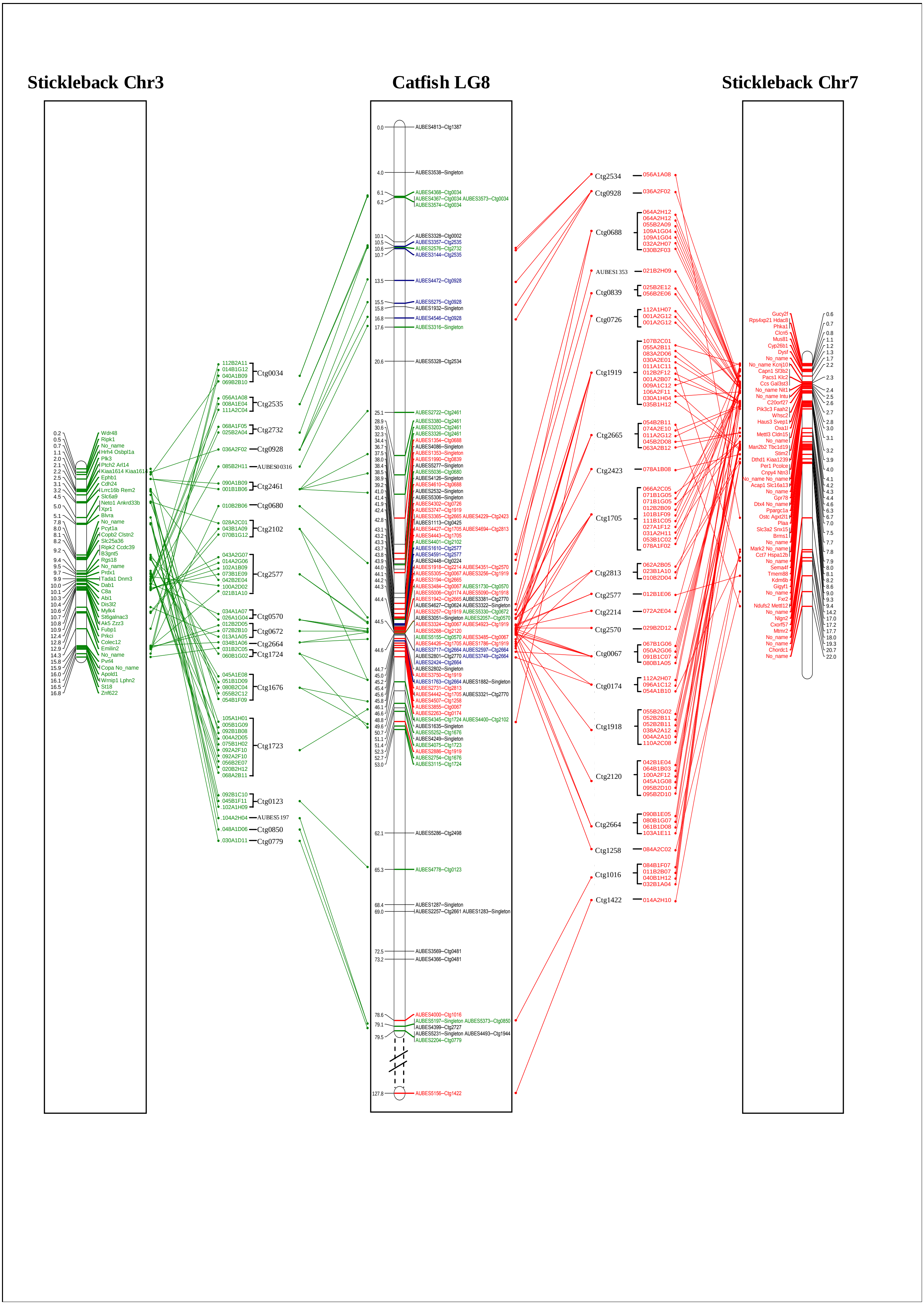
Stickleback Chr3
Catfish LG8
Stickleback Chr7
Ctg0034
Ctg2535
Ctg2732
Ctg0928
AUBES0316
Ctg2461
Ctg0680
Ctg2102
Ctg2577
Ctg0570
Ctg0672
Ctg2664
Ctg1724
Ctg1676
Ctg1723
Ctg0123
AUBES5197
Ctg0850
Ctg0779
Ctg2534
Ctg0928
Ctg0688
AUBES1353
Ctg0839
Ctg0726
Ctg1919
Ctg2665
Ctg2423
Ctg1705
Ctg2813
Ctg2577
Ctg2214
Ctg2570
Ctg0067
Ctg0174
Ctg1918
Ctg2120
Ctg2664
Ctg1258
Ctg1016
Ctg1422
