# Supplementary material for: Comparative genomic analysis of catfish linkage group 8 reveals two homologous chromosomes in zebrafish and other teleosts with extensive inter-chromosomal rearrangements
Source: BMC Genomics. 2013 Jun 10;14:387. doi: 10.1186/1471-2164-14-387 (PMC3691659; doi:10.1186/1471-2164-14-387)
Supplement: Additional file 18 — Comparative map between catfish LG8 and green-spotted pufferfish chromosome 15, chromosome 20 and chromosome 6. [file 1471-2164-14-387-S18.pdf]

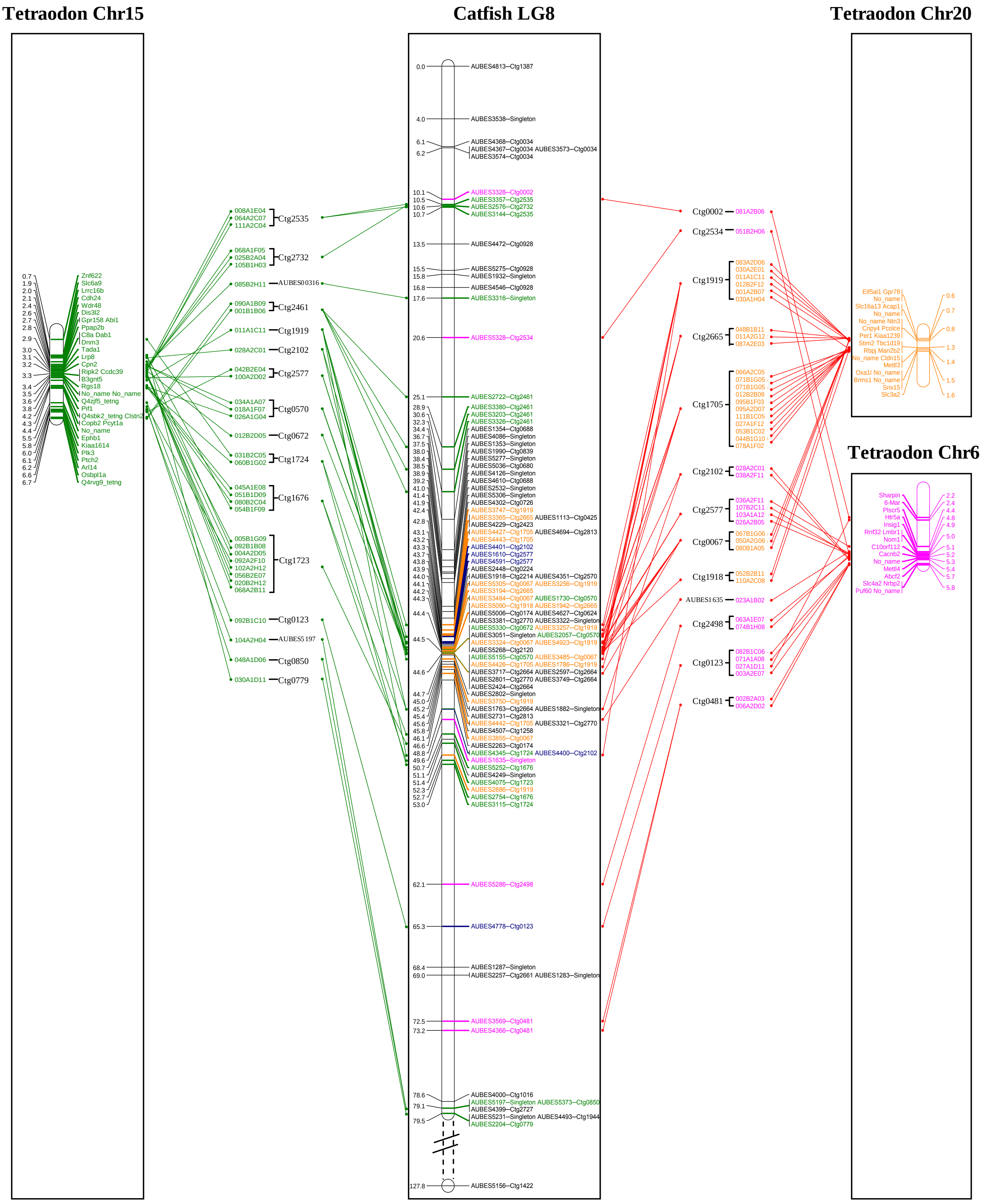

Tetraodon Chr15
Catfish LG8
Tetraodon Chr20
Tetraodon Chr6
Znf622
Slc6a9
Lrrc16b
Cdh24
Wdr48
Dis3l2
Gpr158 Abi1
Ppap2b
C8a Dab1
Dnm3
Tada1
Lrp8
Cpn2
Ripk2 Ccdc39
B3gnt5
Rgs18
No_name No_name
Q4zjf5_tetng
Pif1
Q4sbk2_tetng Clstn2
Copb2 Pcyt1a
No_name
Ephb1
Kiaa1614
Plk3
Ptch2
Arl14
Osbpl1a
Q4rvg9_tetng
Ctg2535
Ctg2732
Ctg2461
Ctg1919
Ctg2102
Ctg2577
Ctg0570
Ctg0672
Ctg1724
Ctg1676
Ctg1723
Ctg0123
Ctg0850
Ctg0779
AUBES0316
AUBES5197
Ctg0002
Ctg2534
Ctg2665
Ctg1705
Ctg0067
Ctg1918
AUBES1635
Ctg2498
Ctg0481
AUBES4813–Ctg1387
AUBES3538–Singleton
AUBES4368–Ctg0034
AUBES4367–Ctg0034 AUBES3573–Ctg0034
AUBES3574–Ctg0034
AUBES3328–Ctg0002
AUBES3357–Ctg2535
AUBES2576–Ctg2732
AUBES3144–Ctg2535
AUBES4472–Ctg0928
AUBES5275–Ctg0928
AUBES1932–Singleton
AUBES4546–Ctg0928
AUBES3316–Singleton
AUBES5328–Ctg2534
AUBES2722–Ctg2461
AUBES5286–Ctg2498
AUBES4778–Ctg0123
AUBES1287–Singleton
AUBES2257–Ctg2661 AUBES1283–Singleton
AUBES3569–Ctg0481
AUBES4386–Ctg0481
AUBES4000–Ctg1016
AUBES5197–Singleton AUBES5373–Ctg0850
AUBES4399–Ctg2727
AUBES5231–Singleton AUBES4493–Ctg1944
AUBES2204–Ctg0779
AUBES5156–Ctg1422
Eif5al1 Gpr78
Slc16a13 Acap1
Cnpy4 Pcolce
Per1 Kiaa1239
Stim2 Tbc1d19
Rbpj Man2b2
Oxa1l No_name
Brms1 No_name
Sharpin
Plscr5
Htr5a
Insig1
Rnf32 Lmbr1
Nom1
C10orf112
Cacnb2
Abcf2
Slc4a2 Nrbp2
Puf60 No_name
